# The Effects of a Multidomain Lifestyle Intervention on Brain Function and Its Relation With Immunometabolic Markers and Intestinal Health in Older Adults at Risk of Cognitive Decline: Study Design and Baseline Characteristics of the HELI Randomized Controlled Trial

**DOI:** 10.2196/69814

**Published:** 2025-10-15

**Authors:** Mark R van Loenen, Lianne B Remie, Mara PH van Trijp, Michelle G Jansen, José P Marques, Jurgen AHR Claassen, Ondine van de Rest, Yannick Vermeiren, Nynke Smidt, Sietske AM Sikkes, Kay Deckers, Marissa D Zwan, Wiesje M van der Flier, Sebastian Köhler, Wilma T Steegenga, Joukje M Oosterman, Esther Aarts

**Affiliations:** 1 Donders Institute for Brain, Cognition and Behaviour, Radboud University Nijmegen, Nijmegen, The Netherlands; 2 Donders Centre for Cognitive Neuroimaging, Donders Institute for Brain, Cognition and Behaviour, Radboud University Nijmegen, Kapittelweg 29, Nijmegen, 6525 EN, The Netherlands, 31 24 361 0750; 3 Division of Human Nutrition and Health, Wageningen University and Research, Wageningen, The Netherlands; 4 Department of Geriatrics, Radboud University Medical Center, Radboudumc Alzheimer Center, Nijmegen, The Netherlands; 5 Department of Epidemiology, University of Groningen, Groningen, The Netherlands; 6 Alzheimer Center Amsterdam, Neurology, Vrije Universiteit Amsterdam, Amsterdam, The Netherlands; 7 Amsterdam Neuroscience, Neurodegeneration, Amsterdam, The Netherlands; 8 Department of Clinical, Neuro and Developmental Psychology, Vrije Universiteit Amsterdam, Amsterdam, The Netherlands; 9 Department of Psychiatry and Neuropsychology, Alzheimer Centrum Limburg, Maastricht University, Maastricht, The Netherlands; 10 Epidemiology & Data Science, Vrije Universiteit Amsterdam, Amsterdam, The Netherlands

**Keywords:** lifestyle, cognitive ageing, risk factors, risk reduction, multidomain, intervention, randomized controlled trial, brain, gut-brain, magnetic resonance imaging

## Abstract

**Background:**

Studies of multidomain lifestyle interventions show mixed results on preventing or delaying cognitive decline in aging. A better understanding of central and peripheral mechanisms underlying these interventions could help explain these mixed findings.

**Objective:**

The HELI (Hersenfuncties na LeefstijlInterventie) study aims to investigate the brain and peripheral mechanisms of a multidomain lifestyle intervention in older adults at risk of cognitive decline.

**Methods:**

The HELI study is a 6-month multicenter, randomized, controlled multidomain lifestyle intervention trial powered to include 104 Dutch older adults at risk of cognitive decline. Individuals were deemed at risk when scoring ≥2 points on a lifestyle-modifiable risk factor scale (eg, overweight, physical inactivity, hypertension, and hypercholesterolemia). The intervention consisted of 5 domains (diet, physical activity, stress management and mindfulness, cognitive training, and sleep) and participants were randomized to one of two groups: (1) a high-intensity coaching group with weekly supervised online and on-site group meetings, exercises, and lifestyle-specific course materials, and (2) a low-intensity coaching group receiving general lifestyle health information sent through email every 2 weeks. The primary study outcomes are changes between baseline and 6-month follow-up in (1) brain activation in dorsolateral prefrontal cortex (dlPFC) and hippocampus and task accuracy during a functional magnetic resonance imaging (fMRI) working memory task, (2) arterial spin labeling-quantified cerebral blood flow in dlPFC and hippocampus, (3) systemic inflammation from blood plasma (interleukin-6, tumor necrosis factor-α, high-sensitivity C-reactive protein) and (4) microbiota profile from feces (gut microbiome diversity [Shannon and phylogenetic diversity] and richness [Chao1]). In addition, we will investigate intervention-induced gut-immune-brain links by assessing relations between effects in primary brain and gut outcomes. Secondary study outcomes include (1) structural and neurochemical magnetic resonance imaging (MRI), (2) anthropometric measurements, (3) neuropsychological test battery scores, (4) lifestyle-related questionnaire and smartwatch measures, and peripheral measures from (5) fecal, (6) blood, and (7) breath analyses.

**Results:**

This work was supported by a Crossover grant (Maintaining Optimal Cognitive Functioning In Aging [MOCIA] 17611) of the Dutch Research Council (NWO), granted in December 2019. The MOCIA program is a public-private partnership. Between April 2022 and October 2023, we successfully included 102 older Dutch adults (mean age 66.6, SD 4.3 years; 67/102, 65.7% female) with ≥2 lifestyle-modifiable risk factors of cognitive aging (median risk 3, IQR 2-3). The most common self-reported lifestyle-modifiable risk factors at baseline were overweight or obesity (76/102, 74.5%), followed by hypertension (58/102, 56.9%), hypercholesterolemia (57/102, 55.9%), and physical inactivity (57/102, 55.9%).

**Conclusions:**

The HELI study aims to enhance our understanding of the working mechanisms of multidomain lifestyle interventions through its comprehensive characterization of central and peripheral markers. We intend to achieve this aim by assessing lifestyle intervention-induced changes in functional and structural MRI brain measures, as well as peripheral measures of the gut-immune–brain axis involved in cognitive aging.

## Introduction

### Background

Multidomain lifestyle interventions have great potential to delay cognitive aging, but the effects of previous trials are small and mixed. Furthermore, previous studies mainly focused on cognitive functioning as the primary outcome. Consequently, underlying neurocognitive, central mechanisms, and involved peripheral pathways remain poorly understood. The HELI (Hersenfuncties na LeefstijlInterventie) study is a 6-month multidomain lifestyle intervention in older adults at risk of cognitive decline based on modifiable lifestyle factors, focusing on involved central and peripheral mechanisms by using a broad range of neuroimaging, blood- and gut-related outcome measures. Below, we explain the rationale of our objectives and hypotheses.

### Lifestyle Interventions in Cognitive Aging

In our aging population, the incidence of cognitive decline and incurable neurodegenerative diseases like Alzheimer dementia (AD) and other types of dementia is increasing drastically [1]. A substantial part of the risk factors for dementia, such as obesity, hypertension, type II diabetes, and physical inactivity [2-6], is modifiable by changes in lifestyle. Indeed, observational research shows a relationship between a healthy lifestyle, such as a healthy diet, regular physical and cognitive exercise, not smoking, and maintaining social contacts, with a lower dementia risk [7,8]. As the signs of neurodegenerative diseases, such as amyloid beta pathophysiology and tauopathy in AD, begin decades before the first symptoms become apparent [9,10], paired with the fact that long-term healthy lifestyles show inverse relations with dementia risk, interventions addressing cognitive decline need to start at an early stage [11]. The World Health Organization (WHO) has therefore designated preventive multidomain lifestyle interventions as having the greatest potential to reduce the risk of cognitive decline and dementia within its 2019 guidelines [12].

Previous multidomain lifestyle intervention trials in aging individuals show small but significant effects on both cognitive functioning and dementia risk, as concluded by a meta-analysis from 2022 [13]. Especially when compared to single-domain interventions, multidomain lifestyle programs showed stronger effects [14,15]. However, previous studies are heterogeneous in population characteristics, intervention design, components, and duration and outcome measures. Moreover, when zooming in on individual studies and considering the latest published trials [16,17], the reported effects can be considered mixed. The 24-month Finnish Geriatric Intervention Study to Prevent Cognitive Impairment and Disability (FINGER) study [18] was the first large, longitudinal randomized controlled trial (RCT) in older adults at risk of cognitive decline (n=1260) and found subtle but robust positive effects of a multidomain lifestyle intervention on cognitive functioning. Other lifestyle interventions such as the Prevention of Dementia by Intensive Vascular Care (preDIVA) study (n=2994) [3], Multidomain Alzheimer Prevention Trial (MAPT; n=1680) [19], Japan-Multimodal Intervention Trial (J-MINT; n=531) [16], and AgeWell.de (n=1030) [17] did not find differences in cognition or dementia risk. However, specific subgroups within these trials with higher dementia risk based on modifiable cardiovascular lifestyle factors [3,19] did show improvements in cognitive functioning. Altogether, these findings point to specific at-risk older adults who are most likely to benefit from multidomain lifestyle interventions.

Crucially, these previous lifestyle intervention studies all had behavioral cognitive tests or dementia incidence as primary outcomes. We still have a limited understanding via which brain mechanisms and potential underlying peripheral pathways, the multidomain lifestyle interventions might exert their effects on cognition. To elucidate underlying brain mechanisms, different neuroimaging techniques are essential to analyze brain health and function in vivo in a longitudinal, intervention setting. Moreover, to understand involved peripheral pathways, we need extensive measures of inflammatory, metabolomic, and microbiome markers in blood and feces.

Among the previous lifestyle intervention studies, only FINGER (n=132, 2 years) and MAPT (n=68, 6 months) used (structural) neuroimaging in a subsample of participants. Within FINGER, no structural (ie, anatomical) differences were found between intervention groups [20], whereas within MAPT, the lifestyle intervention group showed decreased deformation of periventricular areas and temporal lobes, and decreased whole-brain atrophy compared to placebo [19]. In addition, positron emission tomography (PET) scans within MAPT also demonstrated elevated levels of cerebral glucose metabolism within, for example, the hippocampus and the adjacent parahippocampal gyrus [21]. These limited and heterogeneous neuroimaging findings highlight the poor understanding we currently have of underlying neurocognitive mechanisms of lifestyle changes. In addition, some of the previous studies did assess blood, cardiovascular [3], inflammatory [19], or metabolomic markers [18,19], which confirmed that these pathways are influenced by lifestyle changes. However, none of the preceding lifestyle interventions have combined extensive blood and fecal measures to fully understand underlying peripheral(-central) mechanisms.

Therefore, within the HELI study, we will combine a broad neuroimaging protocol, including task-related functional magnetic resonance imaging (fMRI) and quantitative imaging of cerebral blood flow (CBF) as primary central outcomes, with peripheral measures in blood and feces, including blood inflammatory profile and fecal microbiota composition as primary peripheral outcomes. Below, we will explain the rationale of our chosen primary and secondary outcome measures.

### Aging Brain and Lifestyle

The longitudinal process of aging impacts multiple facets of the brain, causing changes in both brain structure and function and, subsequently, in cognition. Age-related structural brain changes are characterized by changes in gray and white matter volume, leading to loss of overall brain volume [22]. Importantly, certain brain structures such as the hippocampus and dorsolateral prefrontal cortex (dlPFC) appear to shrink at a faster rate compared to other brain regions, possibly explained by their increased vulnerability to vascular risk factors such as hypertension and hypercholesterolemia [23-25]. These findings indicate a possible link between lifestyle-modifiable cardiovascular risk factors and aging-related cognitive decline. In addition, the large interindividual differences reported in age-related structural and functional brain deterioration have been proposed to be partially explained by differences in adherence to healthy lifestyle patterns [26].

Earlier studies in pathological aging, specifically in mild cognitive impairment (MCI) and AD, have broadly established key patterns of functional brain deterioration, even in prodromal stages of disease before structural deterioration becomes apparent [27]. These findings indicate that measures of brain function may be more sensitive to interventions targeting age-related cognitive decline and are more strongly modifiable by nature as opposed to brain structure. The most notable cognitive changes associated with normal and pathological aging can be found in the more complex cognitive domains, such as executive functioning and working memory [28]. Working memory is a fundamental cognitive process, supporting higher-order cognitive functions such as planning, problem-solving, and decision-making. Although the overall effects of age on working memory, which shows gradual decreases with advancing age, have been well-established, multiple studies have also identified increased activity in regions of the prefrontal cortex (PFC) in older adults who show comparable working memory performance to young adults [29,30]. These studies indicate that in aging, differences in functional activation patterns, measured with fMRI, may already show a—perhaps compensatory—reorganization before detriments in cognitive performance are observed [28,31]. However, as current literature remains scarce, it remains unclear how this potential functional reorganization is affected by a multidomain lifestyle intervention, especially in at-risk older adults. Another brain function measure, which is a potential cause underlying age-related decline in cognitive functioning, is decreased (local) CBF [32-35]. Multiple studies have reported the predictive value of quantified CBF on cognitive performance in both normal and pathological cognitive aging [32-35]. Moreover, increased local CBF in specific brain regions, such as the hippocampus and PFC, has been directly linked to increased cognitive performance [32]. Although previous studies have found short-term positive effects on CBF after lifestyle improvements such as improved diet or increased physical exercise, it remains unclear whether these effects retain after a multidomain lifestyle intervention and how the increase in regional CBF translates to cognitive functioning in aging [36].

Other promising brain measures in cognitive aging research are the quantification of local iron deposition and neuroinflammation. The accumulation of iron across the lifespan (especially in the deep gray matter), although conventionally found in healthy aging adults [37-39], has been directly linked with increased local oxidative stress leading to neurodegeneration [40-43] and decreased cognitive functioning in older adults with amyloid-β pathology [44]. Similarly, and in addition to intracranial iron deposition, neuroinflammation contributes to neurodegenerative processes in the normally aging brain [45], for example, by affecting the cerebrovascular endothelium leading to neurovascular dysfunction [46]. These impairments could eventually contribute to decreased brain functioning and cognitive decline [47]. We now know that increased regional neuroinflammation—for example, measured by quantifying intracranial myo-inositol concentrations with magnetic resonance spectroscopy (MRS)—is strongly associated with memory dysfunction in normal aging [48,49], in MCI and in AD [49,50]. These findings emphasize the effect of local neurodegenerative effects on specific cognitive domains associated with cognitive aging and dementia.

We currently know that age-related structural and functional brain changes consequently cause cognitive decline, and that brain perfusion, neuroinflammation, and iron accumulation are possible underlying mechanisms. A small number of lifestyle intervention studies, most of which target one specific domain, have reported positive associations between improved diet, exercise, and cognitive training, and neuroimaging measures of cerebral perfusion [51-53] and functional connectivity [54]. However, it is currently unknown to what extent a multidomain lifestyle intervention influences the involved mechanisms of age-related changes in brain activation. Applying a broad neuroimaging protocol could provide important insights into the effects of multimodal lifestyle improvements on the underlying brain mechanisms of age-associated cognitive decline and provide direct mechanistic targets for future interventions.

### Gut-Brain Axis and Peripheral Mechanisms

**Figure 1.**
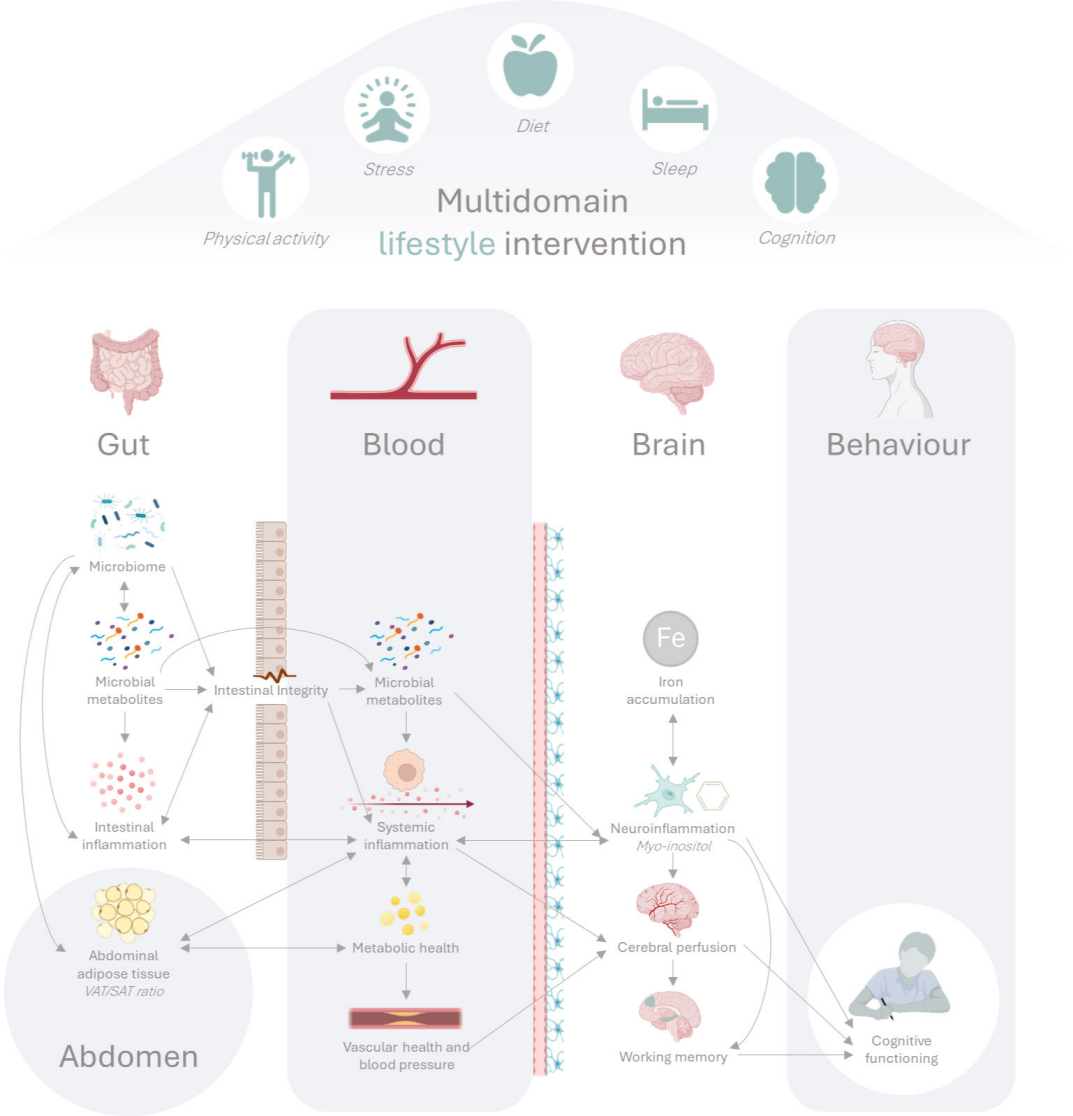
Hypothesized peripheral pathways from gut to brain affecting cognitive functioning, based on the existing evidence mentioned in the introduction. VAT/SAT ratio: ratio of visceral to subcutaneous adipose tissue.

Growing evidence indicates an important role for intestinal health and other peripheral factors, like inflammation, metabolic, and vascular health, in the development of cognitive decline [55,56]. Furthermore, a substantial part of lifestyle effects on brain functioning is expected to arise via mechanisms in the periphery. An overview of these hypothesized pathways and the link with brain health can be found in Figure 1. These pathways are expected to be influenced by multidomain lifestyle interventions at different levels. Below, we will describe the rationale behind these pathways.

The gut microbiome, in particular, has an increasingly recognized impact on brain functioning and behavior via different routes captured in the gut-brain axis. These include the modulation of immune signaling to the brain, affecting the hypothalamic—pituitary—adrenal axis, and transmission via the vagus nerve [57]. Microbiota-derived bioactive metabolites, such as short-chain fatty acids (SCFAs), tryptophan metabolites, (endo)toxins, and secondary bile acids, could mediate the effects of the gut microbiome on brain health [58]. They reflect microbiome function [59] and have demonstrated health effects on the host (eg, [anti]inflammatory and metabolic processes) [60,61], also in aging [62].

We know that aging is associated with changes in microbial composition and diversity and gut health [63]. Older adults more frequently show gut microbiome dysbiosis (imbalance) compared to younger individuals, potentially due to prolonged exposure to an unhealthy lifestyle [64,65]. Particularly, microbial diversity and uniqueness seem to correlate with aging in Western societies [66]. A lower fecal microbial diversity predicted poorer cognitive function in healthy older adults [67]. Microbial metabolites are also affected by aging, as older adults show reduced fecal total SCFA levels [68]. SCFAs, especially butyrate, are known for their beneficial anti-inflammatory effects [69,70] and have been linked to improved cognition in mice [71,72]. Importantly, these microbial changes become more pronounced in the case of pathological cognitive aging like AD. Compared to healthy age-matched individuals, patients with AD show a decreased microbial diversity and stability over time [73], differences in abundance of individual taxa [56,74], and more often have small-intestinal bacterial overgrowth (SIBO) [75]. In addition, a further decrease in levels of fecal SCFAs and tryptophan metabolites was found, which correlated with cognitive decline [76]. However, as these findings mainly come from cross-sectional studies, causal evidence from intervention studies to provide a stronger link between these gut-brain mechanisms is still limited.

Among the proposed gut-brain pathways, inflammation is assumed to play a pivotal role in brain health. Dysbiosis of the gut microbiome can potentially impair the integrity of the intestinal barrier [77], thereby provoking a local inflammatory reaction that could eventually lead to systemic inflammation [78]. In addition, microbiota dysbiosis can also lead to the dysregulation of abdominal adipose tissue, affecting immune and insulin pathways, promoting systemic inflammation even more [79,80]. Low-grade, systemic inflammation is commonly seen in aging [81] and is characterized by a mild and sustained increase in immune mediators in the systemic circulation. For example, the acute-phase response marker C-reactive protein (CRP) and its major regulators interleukin (IL)-6 and tumor necrosis factor (TNF)-α are increased [81,82]. In turn, low-grade, systemic inflammation due to impaired intestinal health can lead to elevated levels of neuroinflammation [83] and is linked with neurodegenerative processes in the normally aging brain.

Importantly, a direct causal role for the gut-immune-brain axis in aging has been demonstrated in mice using fecal microbial transplantation (FMT) [84-87]. These findings emphasize that microbial modulation in humans might be of significant therapeutic benefit in preventing age-related inflammation and cognitive decline [88]. Altogether, the gut microbiome and its interaction with the host can be considered a crucial modulator of (un)healthy aging, determining the rate of physical and cognitive decline [89].

There are numerous studies showing that gut microbiome composition and immune-metabolic health effects on the host are modified by diet and nutritional components [90-92], including the Mediterranean-type diets [93,94]. In addition, other lifestyle components such as physical activity [95,96], stress [97], and sleep [98,99] are known to affect the microbiota composition and intestinal health, as well as immune, vascular, and metabolic health [100-107]. Importantly, we hypothesize that multiple lifestyle domains can synergistically affect peripheral markers and should therefore be targeted simultaneously. However, it is still largely unclear how gut microbiome, immune, and metabolic factors link to brain health in aging, and which mechanisms are involved. By investigating how lifestyle-induced changes in these peripheral factors relate to changes in brain health, we can make stronger conclusions about the role of these peripheral-brain links in cognitive aging. Moreover, elucidating peripheral-brain mechanisms might also explain individual differences seen in lifestyle interventions for healthy cognitive aging.

### Study Objectives and Hypothesis

We designed the HELI study (derived from the Dutch “HErsenfuncties na LeefstijlInterventie,” meaning “Brain function after lifestyle intervention”) to better understand the neurocognitive effects of a multidomain lifestyle intervention and underlying peripheral (central) mechanisms. The HELI study is a 6-month multidomain lifestyle intervention in older adults at risk of cognitive decline (based on lifestyle-modifiable cardiovascular risk factors) with a high and low-intensity intervention arm. We focus on 5 different lifestyle domains within the intervention period: diet, physical activity, stress management and mindfulness, cognitive training, and sleep. We will assess effects on multiple neuroimaging brain measures (primary: working memory task-related fMRI responses and performance, and quantitative imaging of CBF), peripheral measures related to the gut-immune-brain axis (primary: blood inflammatory profile and fecal microbiota composition), as well as their connections. We propose that, compared to the low-intensity group, the high-intensity group will show greater improvement in central neuroimaging outcomes (eg, fMRI and CBF) as well as peripheral outcomes (eg, blood inflammatory profile and fecal microbiota composition). Moreover, we hypothesize that individual changes in central outcomes correlate with changes in peripheral outcomes, as summarized in Figure 1.

## Methods

### Trial Design

**Figure 2.**
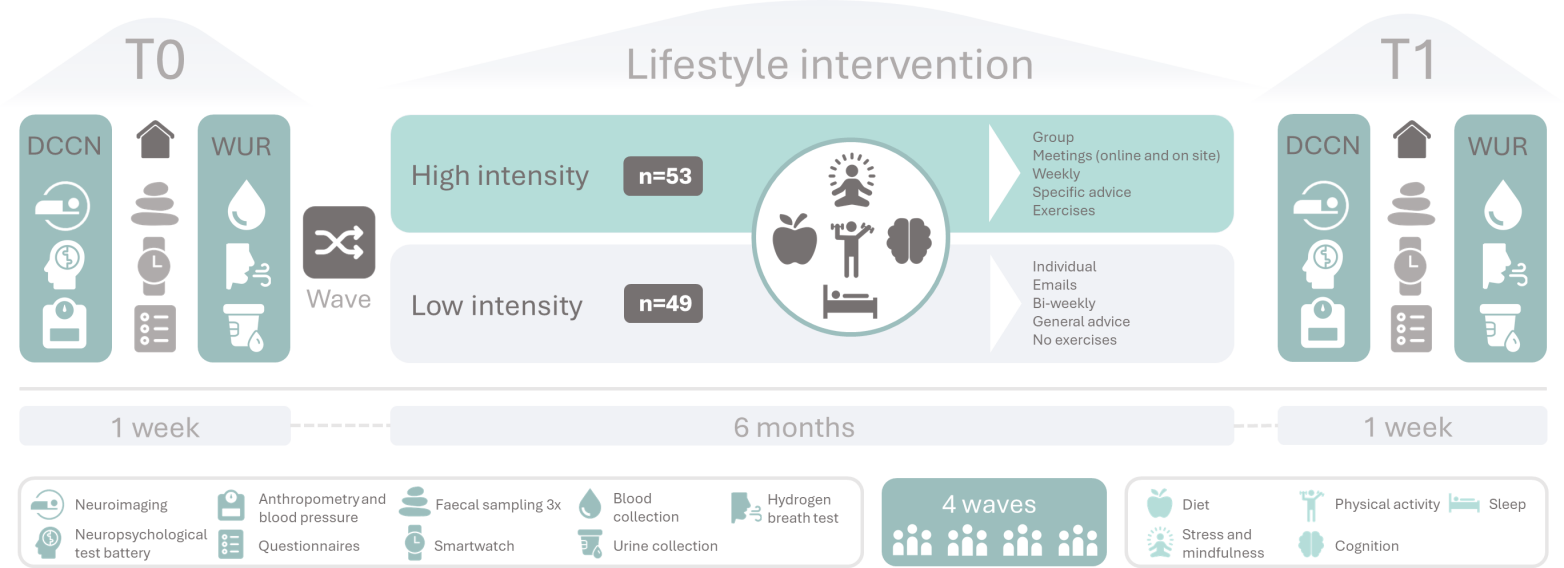
Overview of the study design. Schematic overview of study visits, assessments, and intervention groups. Before **T0 **and after **T1,** the intervention period of 6 months, participants visited the Donders Center for Cognitive Neuroimaging (DCCN) and Wageningen University and Research (WUR) study centers with one week in between.

The HELI study is a multicenter randomized controlled trial, aiming to investigate the effects of a 6-month multidomain lifestyle intervention on brain functioning and its relation with immunometabolic markers in aging. Powered to include 104 total participants, HELI has two parallel-arm treatment groups: (1) a supervised “high-intensity” coaching intervention group with weekly group meetings, lifestyle-related information and exercises, and (2) a nonsupervised “low-intensity” coaching intervention group, receiving information leaflets every 2 weeks. Because the participants of the high-intensity intervention arm were supervised in a group context, we recruited and subsequently randomized participants in 4 different inclusion waves (between May 2022 and October 2023), consisting of 19‐32 participants. Participants partook in outcome measure visits at baseline (T0) and at 6 months follow-up (T1) (see Figure 2).

The HELI study has been reviewed and accepted according to the Medical Research Involving Human Subjects Act (“Wet Medisch-wetenschappelijk Onderzoek met mensen” in Dutch) by an accredited Medical Research Ethics Committee (MREC), the MREC Oost-Nederland on December 2, 2021 (ToetsingOnline filenumber NL78263.091.21, ClinicalTrials.gov ID NCT05777863).

### Methods: Participants, Interventions, and Outcomes

#### Study Setting

The HELI intervention is based on the FINGER-NL lifestyle intervention, a multicenter, randomized, controlled, multidomain lifestyle intervention effectiveness trial in the Netherlands. The rationale and study design of FINGER-NL have been published previously [108]. Both HELI and FINGER-NL are part of the overarching Maintaining Optimal Cognitive Functioning In Aging (MOCIA) research program [109], and follow an inclusion procedure with similar lifestyle modifiable risk factor inclusion criteria as the FINGER study [18,110]. The HELI study is conducted at two separate research centers in the Netherlands, namely the Donders Institute for Brain, Cognition and Behavior, Center for Cognitive Neuroimaging (DCCN; Radboud University, Nijmegen, the Netherlands), and the Division of Human Nutrition and Health at Wageningen University and Research (WUR, Wageningen, the Netherlands). Study visits were performed at both centers, but inclusion, screening, randomization, guidance of the intervention, and monitoring were performed by the DCCN.

#### Recruitment

To achieve adequate participant enrollment and reach the target sample size of 104 included participants, we have used an array of recruitment methods. Our primary sources of enrollments came from: (1) recruitment advertising on Facebook; (2) WUR and DCCN research center participant databases; (3) newsletters of Dutch brain research institutions and associations (eg, Netherlands Brain Foundation and Alzheimer Association Netherlands); (4) messages on WUR and DCCN research center websites and social media channels; (5) articles and recruitment dissemination via flyers, and in local newspapers, radio station, and television broadcast; and (6) word of mouth.

### Eligibility Criteria

#### Inclusion Criteria

Individuals had to meet the following inclusion criteria to be eligible to participate in this study:

1. Aged between 60‐75 years (at the moment of signing written informed consent);
2. Fluency in Dutch (speaking, reading, and writing);
3. Willing to travel to the partaking research centers in Nijmegen and Wageningen (to ensure study center visits are plausible without excessive travel burden);
4. Score ≥2 points on the self-reported “Modifiable cardiovascular risk factor scale” (see Table 1 below), indicating a presently increased risk for cognitive decline based on risk factors which are modifiable by lifestyle changes (adapted from Cardiovascular Risk Factors, Aging, and Incidence of Dementia [CAIDE] [111]).

**Table 1.** Modifiable cardiovascular risk factor scale.

Risk factor	Points
BMI ≥25 kg/m^2^ (overweight or obesity)	1
Physical inactivity (below the 2020 WHO guidelines [112]:<300 minutes of moderate intensity aerobic physical activity, or <150 minutes of vigorous intensity aerobic physical activity per week, spread out over several days)	1
Hypertension (systolic blood pressure ≥140 mmHg, and diastolic blood pressure ≥90 mm Hg)	1 (2 points are assigned if hypertension is not being actively treated with antihypertensive medication, given the increased cardiovascular burden)
Hypercholesterolemia (total cholesterol >5 mmol/L, or LDL-cholesterol >3 mmol/L)	1
Diabetes type II	1
Mild cardiovascular disease (eg, intermittent claudication, varicose veins; In contrast, moderate or severe cardiovascular disease such as stroke, angina pectoris, heart failure, myocardial infarction, or revascularization surgery in the last 12 months before pre-screening are exclusion criteria as described below under section “Exclusion criteria”)	1

#### Exclusion Criteria

Individuals who met any of the following criteria were excluded from participation:

##### Practicalities and Participation

Practical and participation-related factors, of one (or more) of the following:

1. Concurrent participation in other intervention trials;
2. No access to technological infrastructure (ie, computers, smartphones, mobile applications, and internet access) because the intervention is primarily online (eg, online support groups and mobile apps);
3. Cognitive impairment, as determined by a score lower than 23 points on the Telephone Interview for Cognitive Status (TICS-M1) [113], performed during prescreening before inclusion.

##### Clinical Diagnoses and Medical History

Clinical diagnosis by a certified doctor or general practitioner of one (or more) of the following:

1. Cerebrovascular event (eg,transient ischemic attack, cerebral hemorrhage, and stroke);
2. Neurological disease (eg, mild cognitive impairment, multiple sclerosis, Parkinson disease, and epilepsy);
3. Current malignant disease, with or without current treatment;
4. Current psychiatric disorder (eg, depression, psychosis, and bipolar episodes);
5. Symptomatic, moderate to severe cardiovascular disease (eg, stroke, angina pectoris, heart failure, and myocardial infarction);
6. Revascularization surgery in the last 12 months at the moment of prescreening;
7. Inflammatory bowel disease (characterized by diarrhea);
8. Visual impairment, which is uncorrectable with visual aids (eg, total or partial blindness);
9. Hearing or communicative impairment, which is uncorrectable with hearing aids;

##### Magnetic Resonance Imaging Safety–Specific Criteria

Contraindications for magnetic resonance imaging (MRI), of one (or more) of the following:

1. Metal objects located in the upper body (exceptions: tooth fillings and dental crowns);
2. Metal splinters in the body, in particular within the eyes (eg, through labor work in the metal industry);
3. Jewelry items or piercings that cannot be taken off;
4. Undergone brain surgery in the past;
5. Active implants within the body (eg, pacemaker, neurostimulator, internal insulin pump, internal hearing aids that cannot be removed);
6. Medical plasters or patches which cannot or may not be taken off (eg, nicotine patch);
7. Self-reported claustrophobia.

### Interventions: Description

**Figure 3.**
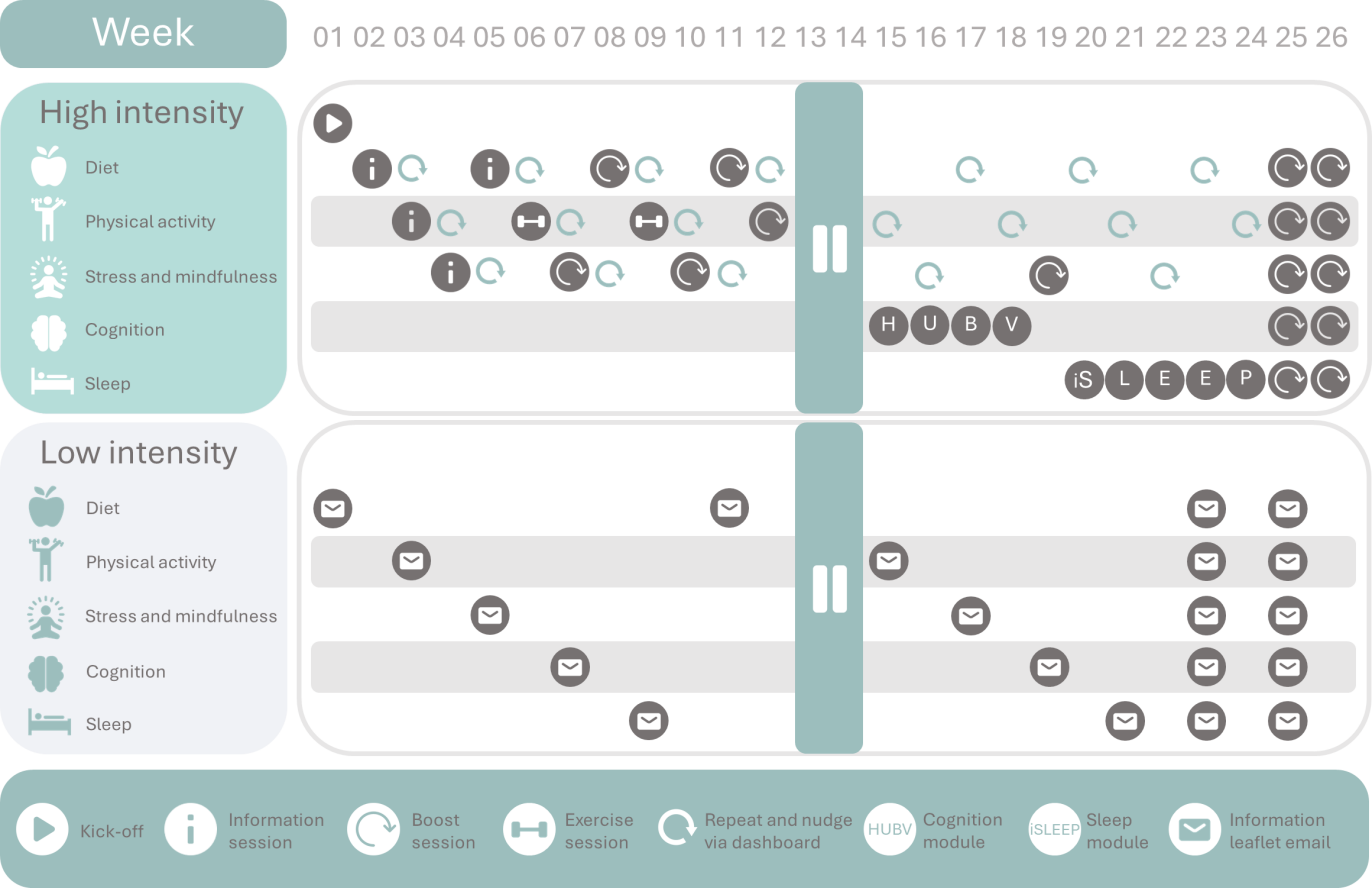
Intervention overview. HUBV (Houd uw Brein Vitaal; “Keep your brain fit”) and iSLEEP are validated psychoeducational and cognitive behavioral therapy programs.

In the HELI multidomain lifestyle intervention, five distinct lifestyle domains were combined, namely (1) diet, (2) physical activity, (3) stress management and mindfulness, (4) cognitive training, and (5) sleep. The intervention period was 6 months (26 weeks) in total, which was subsequently followed by the follow-up outcome measure visits (see Figure 3 for an overview).

The high-intensity intervention group received a structured and personalized intervention program consisting of weekly group sessions, active guidance from an intervention supervisor, and lifestyle domain–related individual and group exercises. The low-intensity intervention group received access to general lifestyle-related health information in an individual context, provided through information leaflets sent by email once every 2 weeks. To promote intervention adherence and blinding, we adopted the FINGER-NL [108] and US POINTER approach [114] of framing both groups as lifestyle intervention recipients, instead of using the label “control group.” The groups differ in intervention structure, engagement, and intensity rather than treatment versus control.

During communication with participants, we refrained from using the terms “high-intensity” and “low-intensity” to promote group blinding as much as possible. Instead, the intervention arms were called “weekly group sessions” for the high-intensity intervention group, and “independently working on your lifestyle” for the low-intensity intervention group. An extensive description and overview of the multidomain lifestyle intervention groups is provided below and depicted in Figure 3.

#### High-Intensity Group

##### Design

In order to achieve the domain-specific goals and ambitions, we provided participants within the high-intensity group with three key intervention elements: (1) weekly online or on-site group sessions to provide information, exercises, and personalized advice, (2) an extensive information folder containing supporting lifestyle-related information, exercises, and relevant practical information, and (3) access to an online dashboard with a personal intervention environment. Each weekly group session took approximately 90 minutes. We expected that participants required an average of 90 minutes to put additional work in the provided lifestyle advices, exercises, and other practical aspects (eg, reading online dashboard and intervention information folder; see S3.1 in Multimedia Appendix 1), resulting in a weekly expected intervention effort of 180 minutes.

##### Group Sessions

The weekly group sessions were offered throughout the 6-month (26 weeks) intervention period. For 20 out of 26 weekly sessions, a specific lifestyle domain acted as the central theme for a given week (Figure 3). At week 1, after the baseline measurements of all participants in a wave and subsequent randomization had been concluded (T0), the intervention started off with an on-site introductory session.

The weekly group sessions provided participants with detailed lifestyle-related information, guidelines, exercises, and advice, as well as the opportunity for discussion with other group members and with the intervention supervisor. Weekly group sessions were guided by the intervention supervisor and were composed of the following recurring elements: social interaction, introductory question round, reiteration of previous lifestyle domains, dissemination, exercises, and a final question round (see Table S1 in Multimedia Appendix 1 for further details).

Over the 6-month intervention period, each of the five lifestyle domains was addressed in separate weekly group sessions (see Figure 3). We provided three distinct types of weekly group sessions:

- “Information sessions” primarily focused on giving general instructions, advice, and exercises on a particular domain. During information sessions, participants were encouraged to ask questions and share experiences, problems, or advice with the group.
- “Boost sessions” primarily focused on recalling and reiterating domains which had been discussed in previous information or booster sessions, whilst also providing smaller amounts of new information. The main goal of boost sessions was to keep participants engaged in the multiple different lifestyle domains throughout the 6-month intervention period and remind them of instructions, advice, and exercises from previous sessions. During boost sessions, more time was reserved for repeating previously discussed material and information, group discussions, and sharing experiences, advice, or problems regarding one or more specific domains.
- “Exercise sessions” primarily focused on explaining and performing muscle-strengthening exercises (full-body work-out, ie, including all major muscle groups) in combination with balancing and stretching exercises in a group setting at an exercise center under the supervision of a physical exercise instructor specialized in instructing and guiding older adults. The exercise instructor provided instructions for conducting these exercises and for preventing strains or injuries.

Domains that were expected to require more instructions and guidance from the start (ie, diet and physical exercise) have two information sessions and 2 boost sessions or one information session and 2 exercise sessions, whilst domains that required less initial group and personal guidance and instruction (ie, stress management and mindfulness) had one information session and three boost sessions. The lifestyle domains of cognitive training and sleep differed from the information and boost session design, as these domains were composed of a psychoeducational program for the domain of cognition (spanning four consecutive weeks) and a sleep course module for the domain of sleep (spanning five consecutive weeks), further explained below. A more detailed explanation of the ambitions, goals, and content of the 5 lifestyle domains within the HELI lifestyle intervention is provided below and in S3.1 in Multimedia Appendix 1.

### Ambition and Goals of Lifestyle Domains

#### Diet

##### Ambition and Goals

The goal of the diet domain was to support participants to adhere to the Mediterranean-DASH Intervention for Neurodegenerative Delay (MIND) diet, a diet specifically designed to slow down aging-related cognitive decline [115], and the Dutch guidelines for a healthy diet [116] which is associated with protective effects on multiple major (chronic) diseases (eg,coronary heart disease, stroke, diabetes, heart failure, and dementia). Specifically, we use the MIND-NL diet, which is adapted to the Dutch context and serving sizes [117]. Briefly, the MIND-NL diet-specific food recommendations comprise (1) green leafy vegetables, (2) other vegetables, (3) berries and strawberries, (4) whole-grain products, (5) unsalted nuts, (6) (unfried) fish, (7) (unfried) poultry, (8) beans and legumes, and (9) olive oil. MIND-NL diet-specific food restrictions comprise (1) full-fat cheese; (2) butter and stick margarine; (3) red and processed meat; (4) take-out, fried foods, and snacks; (5) cookies, pastries, and sweets; and (6) wine and alcoholic beverages [115]. The Dutch guidelines for a healthy diet consist of adhering to a healthy diet of the five major healthy food groups (in Dutch: “de Schijf van Vijf”): (1) fruit and vegetables; (2) (unsaturated) oil and spreads; (3) dairy, nuts, fish, legumes, meat, egg, or alternatives; (4) (whole-grain) bread, grain products and potatoes; and (5) hydration (water, tea, and coffee). As an additional component to this domain, to comply with Dutch national recommendations for older adults [118,119], participants are advised to take additional vitamin D_3_ supplements throughout the intervention period (see S3.1 in Multimedia Appendix 1 for age and sex-specific advised doses). For more details on the exercises, information, and goals of the diet domain, see S3.1 in Multimedia Appendix 1.

### Physical Activity

#### Ambition and Goals

The goal of the physical activity domain was to support participants to adhere to the official 2020 WHO Physical Activity guidelines [112]. These guidelines recommend engaging in physical activity of moderate intensity (eg, swimming, running, and cycling) for at least 300 minutes per week (spread out over several days), or 150 minutes of vigorous intensity aerobic physical activity (eg, cycling at a fast pace and playing singles tennis), or an equivalent combination of moderate- and vigorous-intensity physical activity (spread out over several days). Moreover, the guidelines recommend performing muscle-strengthening activities at moderate (or greater intensity) that involve all major muscle groups at least twice a week, combined with balance exercises. Finally, the amount of sedentary behavior (eg, sitting and lying down) must be limited according to these guidelines.

### Stress Management & Mindfulness

#### Ambition and Goals

The goal of the stress management and mindfulness domain was to support participants in dealing with daily stress and becoming aware of their own thoughts and feelings, and to support participants in making brain-healthy choices regarding stress management. Participants received a selection of exercises from an evidence-based, self-guided online mindfulness training (provided through the VGZ Mindfulness Coach app [120]), as well as information regarding the potential negative effects of (chronic) stress on the body and brain, as well as practical advice on how to combat stress and improve relaxation.

### Cognitive Training

#### Ambition and Goals

The goal of the cognitive training domain was to inform participants about functional and structural changes in the aging brain, to support participants in learning about strategies that could aid in coping with cognitive changes which are a part of normal aging (eg, informing participants about memory strategies and sharing tips to improve attention capabilities), and how to ultimately apply these strategies in daily life. The cognitive strategy training was based on the Dutch “Houd uw brein vitaal!” (HUBV; “Keep your brain fit!”) psychoeducational program [121]. The HUBV program consists of 2 main parts: lifestyle and memory (complaints), and effective strategy training (see S3.1 in Multimedia Appendix 1 for a more extensive description).

### Sleep

#### Ambition and Goals

The goal of the sleep domain was to support participants in improving sleep quality (and if applicable, reduce insomnia), and to inform participants about the possible health risks associated with poor sleep in aging. Participants received a 5-week sleep counseling module using a validated, guided, online Cognitive Behavioral Therapy for Insomnia (CBT-I), provided via i-Sleep (which was made available on paper to participants) [122,123]. During the program, participants received information about the different stages of sleep, a healthy sleep pattern, how this pattern might change as we get older, and practical advice on how to improve sleep quality. The course consisted of the following modules: psycho-education, sleep hygiene, behavioral interventions (eg, stimulus control and sleep restriction), relaxation, and dealing with worrying or sleep-obstructing thoughts. Specific recommendations and restrictions were also provided to improve sleep quality and quantity, such as a proper diet, physical exercise, and refraining from smoking or using alcohol before bedtime.

### Low-Intensity Group

Participants randomized to the low-intensity intervention group received general lifestyle-related health information, addressing the aforementioned 5 lifestyle domains. Participants received this general information through information leaflets (1‐2A4), through email, once every 2 weeks. The information leaflets discussed the lifestyle domains but exclusively offered general health information without providing participants any individual or personal guidance based on their personal circumstances. For example, participants were generally told that a healthy diet promotes a healthy brain, and which kind of food components are promoted by the guidelines for a healthy diet, but participants were not guided or advised how their own personal diet should therefore be adjusted to fit these diet recommendations and guidelines.

The information of these leaflets was also handed to the high-intensity intervention group to ensure both groups were provided with the same general health advice.

### Outcomes

All outcome measures were measured at baseline (T0) and after participation in the 6-month multidomain lifestyle intervention (T1), during visits at both research centers. Blood drawing and breath tests were performed in a fasted state. Some outcome measures, such as lifestyle domain or adherence-related questionnaires, were also collected throughout the intervention period. For a detailed overview of all outcomes and the collection schedule, see S4 in Multimedia Appendix 1.

### Primary Outcomes

The primary outcomes are the changes between baseline (T0) and follow-up after 6 months (T1) in:

1. Brain activity during working memory in dlPFC [124] and hippocampus (Harvard-Oxford atlas), using fMRI blood-oxygen-level-dependent (BOLD) activity and task accuracy during a numerical N-back task [125];
2. Cerebral perfusion levels, measured by MRI arterial spin labeling in dlPFC and hippocampus;
3. Peripheral immunometabolic biomarker levels in blood plasma (inflammation markers IL-6, TNF-α, hs-CRP) and feces microbiota diversity (Shannon- and phylogenetic diversity) and richness (Chao1).

In addition, we will investigate intervention-induced gut-immune-brain links by assessing the relation between the effects found in the abovementioned primary peripheral markers and brain outcome measures.

### Secondary Outcomes

The secondary outcomes are the changes between baseline (T0) and follow-up after 6 months (T1) in:

1. Structural MRI (eg, gray and white matter volume, ventricular enlargement, abdominal adipose tissue T1 assessment), neurochemical assessment of neuroinflammation in dlPFC and hippocampus (using MRS) and whole brain analyses of primary neuroimaging outcomes (BOLD activity during working memory, cerebral perfusion);
2. Anthropometric measurements (eg, BMI, waist-to-hip ratio) and blood pressure;
3. Cognitive test performance scores of cognitive domains predominantly affected by cognitive aging (executive functioning, working memory, and processing speed) as measured by a neuropsychological test battery (NTB) containing: (1) Rey Auditory Verbal Learning Test (RAVLT) [126], (2) Verbal Fluency Test (VFT) [127], (3) Trail Making Test (TMT) part A&B [128], (4) Digit Symbol Substitution Test (DSST) [129], and (5) Digit Span Test (DST) [129];
4. Lifestyle domain-related questionnaire scores for (1) diet (MIND-adjusted Eetscore [130]), (2) physical activity (Short Questionnaire to Assess Health-enhancing Physical Activity (SQUASH) [131], LASA Sedentary Behavior Questionnaire (SBQ) [132] and SARC-F Sarcopenia Questionnaire [133]), (3) stress management & mindfulness (Perceived Stress Scale (PSS) [134], Hospital Anxiety and Depression Scale (HADS) [135], Five Facet Mindfulness Questionnaire (FFMQ) [136]), (4) cognitive training (Cognitive Failures Questionnaire (CFQ) [137] and Metamemory In Adulthood Questionnaire (MIA) [138]), (5) sleep (Pittsburgh Sleep Quality Index (PSQI) [139]).
5. Physiological parameters measured on the wrist using a smartwatch over a 7-day period (day and night), including galvanic skin response, skin temperature, and heart rate parameters (measured by photoplethysmography sensing), and contextual motion parameters (measured by an integrated 3-axis accelerometer and 3-axis gyroscope);
6. Fecal analysis, including intestinal transit time [140], stool water content, pH, microbiota composition (both relative- and absolute abundances), metabolite profiles, and inflammatory biomarkers;
7. Blood analysis, including intestinal integrity markers, inflammatory markers, cardio-metabolic and oxidative stress markers, nutritional status, (early) AD markers, and dietary- and microbiota-derived metabolites;
8. Breath analysis to measure SIBO [141,142].

### Other Study Parameters

Other study outcomes include the following:

1. Baseline demographics (age, sex, education level, ethnicity, etc) and medication history;
2. Baseline intracranial myelin and iron deposition measured by quantitative susceptibility mapping (QSM) MRI, and by relaxation rates (R_1_ and R_2_*– longitudinal and apparent transverse relaxation rates respectively);
3. Baseline IQ based on the Dutch Adult Reading Test (DART) [143];
4. Intervention adherence (in the high-intensity group) during the intervention period: group meeting attendance and completion of provided exercises;
5. Changes between baseline (T0) and follow-up after 6 months (T1) of the Montreal Cognitive Assessment (MoCA) [144];
6. Urine analysis of microbiota-derived bioactive compounds (changes between baseline (T0) and follow-up after 6 months (T1);
7. Changes between baseline (T0) and follow-up after 6 months (T1) in other health or lifestyle-related questionnaire scores, including (1) (psychosocial) complaints in daily life (Dutch Four Dimensional Complaint Questionnaire [145]), (2) quality of life (EQ-5D-5L [146]), (3) modifiable dementia risk score (“Lifestyle for BRAin health (LIBRA) [147]), (4) social contact and perceived social support (Lubben Social Network Scale [148]), (5) cognitive coping strategies (Cognitive Emotions Regulation Questionnaire [149]), (6) apathetic symptoms (Starkstein Apathy Scale [150]), (7) gastrointestinal symptoms rating scale (GSRS) [151], and (8) stool consistency (Bristol Stool Scale [152]).

### Sample Size

The expected effect size for this study was determined based on other, mostly single-domain, lifestyle intervention studies with similar outcomes as our primary outcomes in the brain (eg, cerebral perfusion, working memory assessments) and in the periphery (eg, immunometabolic biomarkers, gut microbiome diversity). These studies generally found medium-to-large effect sizes (see Table S4 in Multimedia Appendix 1 [52,53,95,106,107,153-161]). Therefore, we deemed a slightly above-medium effect size of 0.30 (Cohen f[V]) suitable for our primary outcomes from baseline (T0=week 0, before start intervention) to follow-up (T1=week 26, after end intervention). The sample size calculation was performed using G*Power (version 3.1.9.6; Universität Kiel [162]). Using a multivariate ANOVA (with repeated measures and within-between interaction) *F* test with a power of 80% and a 2-tailed α-error probability of .05, we calculated that a total of 90 study participants would be required. However, based on our experience with previous fMRI and intervention studies, we expect a potential loss of data of 15% due to poor fMRI data quality (eg, motion artifacts) and potential intervention drop-outs. Therefore, to reach sufficient power, we would require a total sample size of 104 (90 participants×15% potential data loss=103.5=104) participants in this study.

### Methods: Randomization and Blinding

Participants were randomized per inclusion wave, using stratified (2,4) block-randomization to provide a 1:1 allocation between our two intervention arms. Randomization was stratified by the factors sex (male vs female), risk factor profile (medium risk 2‐3 points vs high risk ≥4 points), and a combined factor of education level +age (70+ yearsor low education vs 60‐69 years and high education, specified as university of applied science associate degree (Dutch: HBO associate degree or higher). As with our sample size, (2,4) block-randomization was only possible with up to 3 strata, age, and education were combined in one stratification factor as they are both important covariates of cognitive functioning and aging-related cognitive decline [163].

An independent researcher from the DCCN was authorized to manually perform the randomization. This authorized researcher had no affiliation with the project and therefore had no bias in the allocation procedure.

Given the nature of the intervention arms, the participants and intervention supervisor were unable to remain blinded to the assignment to the intervention arms. Researchers conducting the follow-up outcome measure visits remained blinded and unaware of the intervention arm allocation. Only in case of unforeseen circumstances would deviations be made (eg, an unblinded researcher conducting the MRI protocol). Neuropsychological tests and task trainings (ie, n-back task) were conducted by a blinded researcher in all cases. Participants were asked to refrain from speaking about the intervention contents and experience during the follow-up outcome measure visits to prevent accidental unblinding.

The requirement for emergency unblinding was not applicable to this study, as participants were unblinded to the group allocation and the nature of the study setting.

### Methods: Statistics and Analysis

We will test for change between T0 and T1 in the primary and secondary outcome variables, both within-group and between-group, using linear mixed-effect models with random effects for intercept (individuals). The intervention group and time will be included as fixed effects, as well as the interaction term between intervention group and time, to model the difference in change between T0 and T1 as a function of group allocation.

In addition, we will test for correlations in change (T0 minus T1) between (primary) brain outcomes and (primary) peripheral outcomes. We will select an appropriate correlation test for our data, based on whether assumptions are met. We will include both the high- and low-intensity groups if there is enough variance in change across both groups; else, we will only include the high-intensity group.

The level of significance will be set at 0.05 (2-sided). FDR correction will be used to account for multiple comparisons of secondary and exploratory outcomes. FWE correction will be used for secondary whole-brain analyses.

As part of exploratory analyses to assess the domain-specific effects on our primary outcome measures, we will take the pre- and posteffects of the primary outcome measures and use domain-related questionnaire scores and smartwatch-derived physiological data as a measure of improvement for each respective domain as predictors in linear mixed-effect models.

We will perform an intention-to-treat analysis. Participants who withdrew from the study during the 6-month intervention period, but had not withdrawn consent, were invited to the T1 (after week 26) follow-up outcome assessments. This means that we will use all available collected data, including collected (follow-up) data of participants who dropped out of the intervention. Missing data or data with insufficient quality, however, will not be used in the analysis.

### Ethical Considerations

The HELI study has been reviewed and approved by the MREC Oost-Nederland on December 2nd 2021 (ToetsingOnline filenumber NL78263.091.21, ClinicalTrials.gov ID NCT05777863). All participants provided written informed consent before study inclusion, and participants were free to opt out of participation at any stage of the study. Obtained study data were only accessible to the research team directly involved in the study, and personal privacy was protected by removing personal identifying information from the dataset. Participants received compensation for travel expenses and trial participation, which were evaluated and approved by the MREC to avoid financial motivation in study participation.

## Results

**Figure 4.**
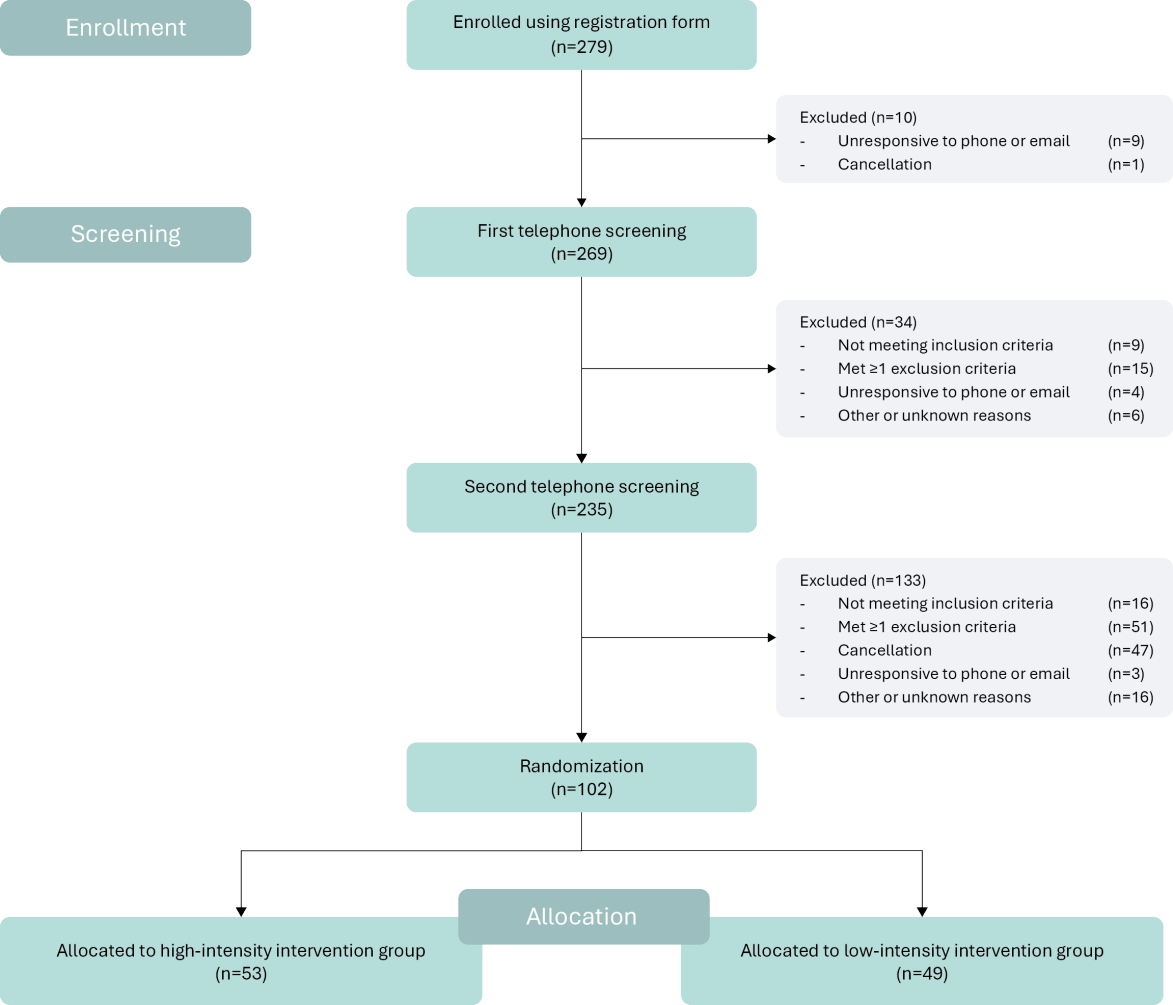
CONSORT (Consolidated Standards of Reporting Trials) study flowchart of HELI (Hersenfuncties na LeefstijlInterventie) study participant enrollment, screening (inclusion and exclusion), randomization, and allocation.

This work was supported by a Crossover grant (MOCIA 17611) of the Dutch Research Council (NWO), granted in December 2019. Recruitment started in April 2022 and concluded in October 2023. Over this period, a total of 279 people showed interest in participating and enrolled, of which 269 participated in the first round of telephone screening and 235 participated in the second round of telephone screening (see Figure 4). We were able to initially include our goal sample size of 104 participants, but 2 last-minute cancellations resulted in a final included sample size of 102 older Dutch adults. This sample size was subsequently randomized in the two parallel intervention arms, with n=49 participants in the “low-intensity” intervention group and 53 participants in the “high-intensity” intervention group.

The baseline measurements at the DCCN and WUR centers were successfully completed by October 2023. The baseline mean age of our sample population was 66.6 (4.3) years, of which 65.7% (n=67) was female (see Table 2). The mean total years of education was 16.2 (SD 4.6) years. At baseline, 9.8% (n=10) of participants had a low level of education (International Standard Classification of Education [ISCED] 0‐2), 34.3% (n=35) had a medium level of education (ISCED 3‐4), and 55.9% (n=57) had a high level of education (ISCED 5‐8). The median risk, measured by points on the self-reported lifestyle-modifiable risk factor scale, was 3 (IQR 2‐3) points. The most common self-reported lifestyle-modifiable risk factors of cognitive aging at baseline were overweight or obesity (76/102, 74.5%), hypertension (58/102, 56.9%), hypercholesterolemia (57/102, 55.9%), and physical inactivity (57/102, 55.9%). See Table 2 for an overview of the HELI study baseline characteristics.

**Table 2.** Baseline characteristics of the HELI (Hersenfuncties na LeefstijlInterventie) study (n=102).

Characteristic	Value	
Participant demographics		
Age (years), mean (SD)	66.6 (4.3)	
Sex (female), n (%)	67 (65.7)	
Educational level, n (%)^a^		
Low	10 (9.8)	
Medium	35 (34.3)	
High	57 (55.9)	
TICS-M1 score, mean (SD)	29.0 (3.6)	
Self-reported inclusion factors		
Overweight and obese (BMI ≥25), n (%)	76 (74.5)	
Obese (BMI ≥30), n (%)	35 (34.3)	
Physically inactive, n (%)^b^	57 (55.9)	
Hypertension, n (%)^c^	58 (56.9)	
Hypertension medication usage, n (%)	84 (82.4)	
Hypercholesterolemia, n (%)^d^	57 (55.9)	
Diabetes type II, n (%)	20 (19.6)	
Mild cardiovascular disease, n (%)^e^	11 (10.8)	
Risk severity		
Risk factor score, median (IQR)	3 (2-3)	

a Based on the International Standard Classification of Education (ISCED 2011) guidelines (low, medium, and high education categorization).

b Based on nonadherence to the WHO Guidelines on physical activity (300 or more min of moderate aerobic activity or 150 min of vigorous aerobic activity per wk).

c Based on systolic blood pressure of ≥140 mm Hg and diastolic blood pressure ≥90 mm Hg.

d Based on total cholesterol >5mmol/L or LDL-cholesterol of >3 mmol/L.

e Based on mild cardiovascular disease not mentioned in exclusion criteria (eg, varicose veins and atherosclerosis).

## Discussion

### Principal Findings

We described the design of the HELI study, an RCT focusing on the mechanisms of a 6-month multidomain lifestyle intervention in Dutch older adults at risk of cognitive decline, by assessing functional and structural MRI-related brain measures as well as peripheral measures related to the gut-immune-brain axis. The study design is based on the design of the FINGER-NL trial [108] and optimized for assessment of mechanisms instead of effectiveness.

The HELI study is characterized by its unique multimodal approach, which enables us to obtain mechanistic insights of a multidomain lifestyle intervention. We are using a broad range of neuroimaging techniques (see Table S3 in Multimedia Appendix 1 for the MRI sequence protocol) and neurocognitive tests to acquire a comprehensive profile of brain health. In addition, we also measure an extensive set of peripheral intestinal and immunometabolic markers, allowing us to investigate gut-immune-brain interactions and discover mediators of lifestyle effects on brain health. Combined with questionnaire data on lifestyle compliance, these brain, blood, and fecal markers have the potential to provide an elaborate overview of the pathways involved in lifestyle effects on cognitive aging and to explain individual differences in effectivity.

Compared to previous 24-month multidomain lifestyle interventions in cognitive aging [3,18,19,164] and the FINGER-NL trial [108], the HELI study intervention of 6 months is significantly shorter. However, our multidomain lifestyle intervention was carefully modified from the 2-year FINGER-NL intervention into a more condensed and intensified 6-month program. Especially, the high-intensity intervention group was characterized by more frequent meetings and group contact, requiring higher weekly effort. Within the field of functional neuroimaging, the duration of HELI (26 weeks) is relatively long compared to other (single-domain) lifestyle intervention studies (6‐9 weeks), which reported significant central mechanistic intervention effects [52,54]. The main focus of our study is on the underlying mechanisms of lifestyle adaptations on cognitive aging, rather than on trial effectiveness. Therefore, we consciously chose to use multiple specific brain and peripheral parameters as primary outcomes, instead of cognitive functioning. We expect these parameters to be more sensitive to lifestyle changes, and therefore to be affected in an earlier stage than cognitive functioning.

We ultimately managed to include a total of 102 participants within our study. Our study population shares a similar baseline lifestyle modifiable risk factor profile (CAIDE) compared with the FINGER study, based on the total number of participants with hypertension, hypercholesterolemia, and diabetes (see Table S5 in Multimedia Appendix 1 [165-167]). Other previous multidomain lifestyle intervention studies in older adults, such as the pre-DIVA, MAPT, and J-MINT studies, used inclusion criteria different from the CAIDE-related lifestyle modifiable risk factors, or included participants solely based on age. Compared with previous studies, our study population is relatively highly educated, and we were unable to include a comparable number of participants with a lower education level. However, many of these earlier studies completed participant inclusion over a decade ago, and education levels have generally increased over time, which may reflect cohort effects. Because education level is often used as a covariate in task-related neuroimaging outcomes and neuropsychological test data, this is an important limitation to consider during data interpretation and comparison of results with other studies. For a more detailed description of the baseline characteristic comparison between the HELI study and previous multidomain lifestyle intervention studies, see Table S5 in Multimedia Appendix 1.

To summarize, the most important strength of the HELI study is its unique combination of multimodal neuroimaging and peripheral phenotyping with a multidomain lifestyle intervention. Moreover, the study has several additional strengths. First, the design has two active arms, which promote blinding and intervention adherence, while preserving between-group comparisons of lifestyle interventions. Second, the group approach within the high-intensity group is a strength, as it encourages peer support and intervention adherence. Finally, the online-onsite combination establishes a future-oriented intervention framework that ensures both quality and broad applicability. Limitations of the study comprise the limited number of objective domain-specific adherence measures and the selection bias toward more highly educated individuals, potentially due to the digital intervention format.

### Conclusions

The HELI study offers a novel mechanistic approach by integrating multimodal neuroimaging and peripheral phenotyping with a multidomain lifestyle intervention in older adults at risk of cognitive decline. Results of the HELI study can help explain the previously observed small effect sizes of multidomain lifestyle interventions to prevent cognitive decline in aging, paving the way for personalized prevention strategies in the future.

## Supplementary material

10.2196/69814 Multimedia Appendix 1 Detailed overview of study recruitment, intervention design, participant timeline, MRI-sequence protocol, sample size calculation, data management, and informed consents.

## Abbreviations

AD: Alzheimer disease
BOLD: blood-oxygen-level-dependent
CAIDE: Cardiovascular Risk Factors, Aging, and Incidence of Dementia
CBF: cerebral blood flow
CBT-I: Cognitive Behavioral Therapy for Insomnia
CFQ: Cognitive Failures Questionnaire
CRP: C-reactive protein
DART: Dutch Adult Reading Test
DCCN: Donders Center for Cognitive Neuroimaging
dlPFC: dorsolateral prefrontal cortex
DSST: Digit Symbol Substitution Test
DST: Digit Span Test
FFMQ: Five Facet Mindfulness Questionnaire
FINGER: Finnish Geriatric Intervention Study to Prevent Cognitive Impairment and Disability
fMRI: functional magnetic resonance imaging
FMT: fecal microbial transplantation
GSRS: Gastrointestinal Symptoms Rating Scale
HADS: Hospital Anxiety and Depression Scale
HELI: Hersenfuncties na Leefstijlinterventie
HUBV: Houd Uw Brein Vitaal
IL: interleukin
ISCED: International Standard Classification of Education
J-MINT: Japan-Multimodal Intervention Trial
LIBRA: Lifestyle for BRAin health
MAPT: Multidomain Alzheimer Prevention Trial
MCI: mild cognitive impairment
MIA: Metamemory In Adulthood
MIND: Mediterranean-DASH Intervention for Neurodegenerative Delay
MoCA: Montreal Cognitive Assessment
MOCIA: Maintaining Optimal Cognitive Functioning In Aging
MREC: Medical Research Ethics Committee
MRI: magnetic resonance imaging
MRS: magnetic resonance spectroscopy
NTB: neuropsychological test battery
PET: positron emission tomography
PFC: prefrontal cortex
preDIVA: Prevention of Dementia by Intensive Vascular Care
PSQI: Pittsburgh Sleep Quality Index
PSS: Perceived Stress Scale
QSM: quantitative susceptibility mapping
RAVLT: Rey Auditory Verbal Learning Test
RCT: randomized controlled trial
SBQ: Sedentary Behavior Questionnaire
SCFA: short-chain fatty acid
SIBO: small-intestinal bacterial overgrowth
SQUASH: Short Questionnaire to Assess Health-enhancing Physical Activity
TICS-M1: Telephone Interview for Cognitive Status
TMT: Trail Making Test
TNF: tumor necrosis factor
VFT: Verbal Fluency Test
WHO: World Health Organization
WUR: Wageningen University and Research
