# Supplementary material for: The Effects of a Multidomain Lifestyle Intervention on Brain Function and Its Relation With Immunometabolic Markers and Intestinal Health in Older Adults at Risk of Cognitive Decline: Study Design and Baseline Characteristics of the HELI Randomized Controlled Trial
Source: JMIR Res Protoc. 2025 Oct 15;14:e69814. doi: 10.2196/69814 (PMC12527369; doi:10.2196/69814)
Supplement: Multimedia Appendix 1 [file resprot-v14-e69814-s001.doc]

# S1. Recruitment: Screening and inclusion

Individuals willing to participate in the HELI study were directed to our online registration form in LimeSurvey (LimeSurvey GmbH, Hamburg, Germany. URL http://www.limesurvey.org). Within the online registration form, an initial pre-screening was included. Only individuals that passed the inclusion- and exclusion criteria pre-screening questions were redirected to the contact form in LimeSurvey, which shared their contact details with the researchers. No data from the pre-screening questions were saved within the survey.

Individuals who registered for the HELI study via the online registration- and contact form were subsequently contacted by phone by one of the study investigators, within 1 week. During this initial phone call, further study details were provided and potential participants had the ability to ask questions. After the phone call, the participant information folder containing extensive information about the study requirements and procedures, and a separate screening informed consent were sent to their provided home address by postal service. After 1-2 weeks, when the potential participant had read the study information, asked remaining questions and returned the signed screening informed consent, the screening informed consent was signed by an involved researcher and a telephonic screening was performed. During this screening by phone, all inclusion- and exclusion criteria were checked with the potential participant to ensure the participant was eligible for participation. In addition, a TICS-M1 (Modified Telephone Interview for Cognitive Status) [1] was performed to rule out participants with possible undiagnosed cognitive impairment. Subsequently, eligible participants were invited for baseline test assessments at the study centres.

A written informed consent for all study procedures was obtained during the first study visit, before randomization or any other study-specific procedures were performed.

# S2. Responsibility for intervention group guidance

For each inclusion wave a primary intervention supervisor was appointed. This supervisor role is carried out by one research team member, and their guidance responsibilities depended on the intervention arm (high-intensity, low-intensity or both). The intervention supervisors (MRvL, LBR, MGJ) remained in close contact with the lifestyle-domain experts of FINGER-NL, who assisted in designing the intervention content, to be able to get direct input (e.g. assistance in answering complex lifestyle-related questions). For the high-intensity intervention arm, the intervention supervisor was responsible for preparing the schedule, organizing and presenting the weekly (online) group meetings, promoting group contact and peer-discussions, supporting and monitoring intervention adherence, discussing intervention-related problems, and addressing lifestyle-related questions from the group. For the low-intensity intervention arm, the intervention supervisor was responsible for sharing the health information periodically by e-mail and addressing lifestyle-related questions from participants by e-mail.

# S3. Intervention

## S3.1 High-intensity intervention

This first session was dedicated to introducing the participants to the intervention supervisor, presenting the concept of the intervention, handing over intervention material (e.g. information booklets), introducing the online dashboard, and registering the accounts necessary to gain access and participate in the online meetings and online dashboard. The online group sessions were hosted on a HELI-specific Microsoft Teams environment, with access to the online group sessions, slides and notes from previous sessions, a Q&A digital bulletin board, and a discussion channel to share personal experiences and advice.

The duration of each element depended on factors such as the needs of a particular group, total group size, or group session topic, but the intervention supervisor made sure that all necessary information for a particular session was communicated to the group. The elements of the group sessions were as follows:

(a) Before the start of the intervention session: as participants are joining the (online) session, the session focused on stimulating social interaction among the participants (e.g. discussing wellbeing).

(b) Introductory question round: participants were able to ask questions regarding all aspects of the intervention, such as practicalities or questions regarding previously covered lifestyle advice, exercises, use of the online dashboard, or questionnaires. Participants were encouraged to share advice and tips with one another.

(c) Reiteration of previous domains: participants received reminders of previously discussed lifestyle domain advice or exercises, or the supervisor started a short discussion about the current progress. The aim of this part of the group session was to remind participants of the advice and guidelines of the multiple different domains, and thereby maintain or improve adherence to all facets of the intervention throughout the entirety of the 6-month intervention period.

(d) Interactive information dissemination session; participants received lifestyle-related advice through an informal lecture-style presentation. Participants were then able to discuss this information with the supervisor and peers.

(e) Exercises; participants received domain-specific exercises to work on during the week after the group session. Exercises were subsequently discussed in the introductory question round of the next group session.

(f) Final question round and closing; participants were able to ask final questions, before the session was concluded.

| **Supplementary Table 1.** Weekly high-intervention group session overview with domain-related session topics | | | |
| --- | --- | --- | --- |
| **Week** | **Domain** | **Session topics** | **Domains reiterated** |
| 1 | - | Intervention introduction, introducing intervention supervisor and group members, registering accounts for applications, receiving IIF. | - |
| 2 | Diet | MIND diet introduction, MIND recommendations and restrictions, healthy diet patterns. | - |
| 3 | Physical activity | Guidelines physical activity, effect of physical activity on brain health, advice on moving more, taking into account safety whilst exercising. | Diet |
| 4 | Stress management & mindfulness | Information on what stress is, and how stress can affect brain health. Importance of relaxation on a healthy stress balance, introduction of mindfulness. | Diet + Physical activity |
| 5 | Diet | Information about reading food packaging labels, practicing making healthy diet choices by comparing different types of labels of similar products. | Physical activity + Stress management & mindfulness |
| 6 | Physical activity | Physical exercise session at exercise-centre (under supervision of physical exercise instructor), performing muscle-strengthening and balance exercises that can also be done at home. | - |
| 7 | Stress management & mindfulness | Discussion about how participants experience stress, and how participants handle stress and relaxation. A number of different mindfulness exercises and techniques are also discussed. | Diet + Physical activity |
| 8 | Diet | Examples of MIND-oriented breakfast, lunch, dinner and snack recipes. Exchange of advice, tips and encountered problems in adhering to the specific MIND recommendations and restrictions. | Physical activity + Stress management & mindfulness |
| 9 | Physical activity | Physical exercise session at exercise-centre (under supervision of physical exercise instructor), performing muscle-strengthening and balance exercises that can also be done at home. | - |
| 10 | Stress management & mindfulness | How does stress affect the way we eat, or exercise? Reiteration of some mindfulness exercises. | Diet + Physical activity |
| 11 | Diet | Interactive MIND quiz with questions regarding the MIND recommendations and restriction guidelines. | Physical activity + Stress management & mindfulness |
| 12 | Physical activity | Interactive physical exercise quiz with questions regarding the physical exercise (in the context of ageing, and the effects of exercising on body and brain health). | Diet + Stress management & mindfulness |
| 13 | - | Two-week break linked to summer or winter holidays | - |
| 14 | - | Two-week break linked to summer or winter holidays | - |
| 15 | Cognitive training | Information about memory, and memory complaints. Discussion about experiences with memory complaints. Exercise introduction of keeping a memory diary. | Diet + Physical activity + Stress management & mindfulness |
| 16 | Cognitive training | Information about external- and internal memory strategies to prevent complaints and decrease forgetfulness in daily life. Memory diary and cognitive training exercises are also discussed. | Diet + Physical activity  + Stress management & mindfulness |
| 17 | Cognitive training | Information about the effect of attention on forgetfulness and memory, finding ways to stimulate the brain, thinking about the future. Cognitive training exercises are also discussed. | Diet + Physical activity + Stress management & mindfulness |
| 18 | Cognitive training | Prospects and goals for the near and distant future with regard to cognitive training are discussed. Memory diary and cognitive training exercises are also discussed. | Diet + Physical activity  + Stress management & mindfulness |
| 19 | Stress management & mindfulness | Recap of mindfulness exercises. Prospects and goals for the near and distant future with regard to stress management and mindfulness are discussed. | Diet + Physical activity |
| 20 | Sleep | Information about the process of sleep (sleep cycles), the importance of sleep and factors that could influence sleep quality and quantity are discussed. | Diet + Physical activity  + Stress management & mindfulness + Cognitive training |
| 21 | Sleep | Information about sleep patterns, and how to create a bedroom for improved sleep quality. The sleep diary and exercises are also discussed. | Diet + Physical activity + Stress management & mindfulness + Cognitive training |
| 22 | Sleep | Information about worrying thoughts, or fears that could impact sleep quality. The sleep diary and exercises are also discussed. | Diet + Physical activity  + Stress management & mindfulness + Cognitive training |
| 23 | Sleep | Information about changing non-helping (negative) thoughts, which could impact sleep quality. The sleep diary and exercises are also discussed. | Diet + Physical activity  + Stress management & mindfulness + Cognitive training |
| 24 | Sleep | Prospects and goals for the near and distant future with regard to cognitive training are discussed. The sleep diary and exercises are also discussed. | Diet + Physical activity + Stress management & mindfulness + Cognitive training |
| 25 | - | Reiteration of all discussed domains. Room for open discussion, and remaining questions. Future goals of adhering to the lifestyle changes are discussed. | - |
| 26 | - | Reiteration of all discussed domains. Room for open discussion, and remaining questions. Future prospects of adhering to the lifestyle changes are discussed. | - |

***Intervention Information Folder***

During the first group session, which is hosted in-person by the intervention supervisor, all participants of the high-intensity intervention group received an Intervention Information Folder (IIF). The IIF contained information about, among other things, the study team and important contact information, time schedule, tutorials, domain-specific information and frequently asked (practical) questions.

***Online dashboard***

Participants within the high-intensity intervention arm were granted access to a personal intervention environment through an online dashboard. This online dashboard was part of the Online Personal Health Environment, of Ivido B.V. (certificates ISO 27001 and NEN 7510; CE-mark risk class I). We used this online dashboard to improve adherence to the intervention, by offering features such as reminders and invitations of online group sessions, or additional lifestyle domain-specific information and exercises. To keep participants engaged and periodically return to the online environment, new notifications were published on a weekly basis on the dashboard environment. After accepting the personal invitation to the HELI-specific dashboard environment, participants were instructed to periodically log-in to the dashboard (e.g. once every 2-3 days) to keep up to date with newly released notifications and features such as lifestyle-related exercises or advice.

*1. Diet*

*Concise description*

Participants were introduced and guided through the MIND diet as well as the Dutch guidelines for a healthy diet. Within the IFF a MIND-diet guide can be found which provides MIND-oriented food recipes for breakfast, lunch, dinner, and healthy snacks. For participants to create their own recipes, whilst adhering to the MIND-diet, information is also provided about reading labels on food packaging.

Apart from providing information, the intervention supervisor also encourages adherence to the MIND diet and Dutch guidelines for a healthy diet by sharing tips and recipes during the sessions and through the online dashboard and IIF. Additionally, the intervention supervisor assists in defining SMART (Specific, Measurable, Achievable, Relevant, Time-bound) formulated goals with respect to dietary change together with (individual) participants. Participants were also advised to take additional vitamin D3 supplements throughout the intervention period. The advised amount of vitamin D3 supplementation in our participant group of 60-75 years old was based on age, sex, exposure to sunlight and skin complexion:

- Men and women over 70 years of age: 20μg/day;
- Women under 70 years of age: 10μg/day;
- Men under 70 years of age, lacking exposure to direct sunlight: 10μg/day;
- Men under 70 years of age, with darker skin complexion: 10μg/day.

*2. Physical activity*

*Concise description*

During the group meetings (see Table 3), information is provided about the importance of being physically active and limiting sedentary behaviour for preserving brain health. Additionally, muscle-strengthening exercises (full-body work-out, i.e. including all major muscle groups) in combination with balancing and stretching exercises are explained and performed in a group setting during two group sessions, at an exercise centre (CleverMove, Wageningen, The Netherlands; [https://www.clever-move.nl](https://www.clever-move.nl/)) under the supervision of a physical exercise instructor specialized in instructing and guiding older adults. The exercise instructor provides instructions for conducting these exercises and for preventing straining or injuries. These exercises, together with keeping up with a more active lifestyle (i.e. taking the bike or walking to do groceries instead of going by car, taking the stairs instead of an elevator, etc.) form the basis of the physical activity domain. Physical activity goals are set at the start of the intervention during the first physical activity domain group sessions and can differ between individuals based on individual level of fitness, regular habits and personal preferences. The exercises for both physical activity and muscle strengthening gradually increase in frequency, intensity and duration over time.

Participants were also offered a mobile application to promote and keep track of daily walking (‘Ommetje’, Netherlands Brain Foundation), online exercise instructions with different difficulty grades suitable for older adults (‘Cordaan Senioren Fitness Gym’, Youtube; ‘Heel Nederland Thuis in Beweging’, MAX Vandaag), and a buddy system to jointly exercise.

*3. Stress management & mindfulness*

*Concise description*

Stress management & mindfulness counselling consists of an evidence-based, self-guided online mindfulness training (provided through the VGZ Mindfulness Coach app) [2]. A selection of exercises from the mobile VGZ Mindfulness Coach app are included in the online dashboard (which simultaneously acts as an active reminder to periodically follow the exercises), such as a 3-minute breathing exercise, body scan, mindfulness theory and visualization and counting of breath. These exercises consist of self-guided (audio) training, with 6 individual online sessions per week with an approximated time investment of 15 minutes each. Once introduced in week 4 of the intervention (see Table 3), the 3-minute breathing exercises from the VGZ Mindfulness Coach app are a regular component to start off the weekly group session throughout the entirety of the rest of the intervention period. Additionally, participants receive information regarding the potential negative effects of (chronic) stress on the body and brain, as well as practical advice on how to combat stress and improve relaxation.

*4. Cognitive training*

*Concise description*

The cognitive strategy training is based on the Dutch ‘Houd uw brein vitaal!’ (HUBV; ‘Keep your brain fit!’) psychoeducational programme [3]. The HUBV programme consists of two main parts: lifestyle & memory (complaints), and effective strategy training (see Table 3). Within the lifestyle & memory (complaints) part, participants receive information about forgetfulness and memory-strategies to incorporate into their lifestyle patterns. Participants are also instructed to keep a memory diary for a number of days. Within the effective strategy training, participants receive information about the importance of attention, information processing, action planning and goal management in order to prevent forgetful behaviour and set goals. Exercises are provided to participants to put this information to practice and incorporate effective strategies into their lifestyle patterns. The HUBV module is concluded with formulating (near and distant) prospects and goals.

*5. Sleep*

*Concise description*

The domain of sleep is addressed in five different group meetings, consisting of five consecutive sessions according to a specialized sleep-therapy programme. The sleep counselling uses a validated, guided, online Cognitive Behavioural Therapy for Insomnia (CBT-I), provided via i-Sleep ([https://www.i-sleep.nl](https://www.i-sleep.nl/); which is made available on paper within the IIF) [4]. For the current study, a version of i-Sleep was created with small textual changes to enhance suitability for the study group (i.e. with a focus on older adults, not solely individuals with insomnia) without changing the actual programme contents. Each of the five individual and consecutive i-Sleep module group sessions provide theory and exercises. Exercises include keeping a sleep diary, and setting and tracking specific bedtimes. Participants are instructed to complete one session of the module (containing information and exercises) every week.

Throughout the entire 5-week programme, participants are asked to keep track of their sleep patterns by means of a daily sleep diary. In week 1, participants receive information about their sleep patterns and habits, and exercises are centred around keeping a sleep diary and answering questions about their current sleep management goals and their current lifestyle patterns. In week 2 and 3, emphasis is put on informing participants about how to improve current sleep patterns (e.g. via sleep restriction, by decreasing worrying thoughts or fear, and decreasing restlessness by finding relaxing activities). Participants are asked to remain logging the sleep diary, and exercises are offered to reflect on possible pitfalls or dangers that could stand in the way of improving current sleep patterns. In week 4, participants are informed about changing non-helping thoughts and their effects on sleep quality, and subsequently receive exercises to practice handling these kinds of thought patterns. In week 5, a summarization of week 1 through 4 is given and goal management is discussed to formulate future plans (and to longitudinally and effectively adhere to new or adjusted sleep patterns if applicable). During the programme, participants additionally receive information about the different stages of sleep, a healthy sleep pattern, how this pattern might change as we get older, and practical advice on how to improve sleep quality (e.g. setting specific times for sleeping, preparing the bedroom, finding relaxation before sleeping by shutting off electronic devices, etc.). Specific recommendations and restrictions are provided to improve sleep quality and quantity, such as proper diet, physical exercise, and refraining from smoking or using alcohol before bedtime.

## S3.2 Intervention: Modifications

All participants in the high-intensity intervention arm received the same lifestyle-related information and exercises. However, the intervention supervisor pursued a personalized approach where each participant was encouraged and guided to more closely adhere to the lifestyle advice of domains which they are personally most interested in.

The low-intensity intervention arm had no personalized approach design. Participants received no additional personal supervision with regard to the information relayed in the biweekly general health information leaflets.

## S3.3 Intervention: Adherence

Improving adherence in order to limit drop-out rate, we utilized efforts to keep participants involved throughout their entire study participation (from baseline outcome measurement scheduling to intervention participation and subsequent follow-up outcome measurement scheduling). The high-intensity intervention group received weekly tips and reminders through the online dashboard, participated in the weekly group meetings and filled in monthly adherence questionnaires as well as a more extensive adherence questionnaire halfway through the intervention, thereby being reminded of the importance to stay on track of the intervention and granting the possibility for the intervention supervisor to identify adherence problems of individual participants throughout the intervention period. The low-intensity intervention group received bi-weekly information leaflets and completed the extensive adherence questionnaire halfway through the intervention. To improve adherence to the outcome visit measurements and prevent last-minute cancellations or no-shows, participants were sent appointment reminders through e-mail and/or by post.

# S4. Participant timeline

Participants underwent assessments at baseline (T_0_) and at follow-up (T_1_) at the two associated research centres with approximately one week in between visits (Error: Reference source not found). In the week between visits, participants were asked to collect faecal samples, wear a smartwatch and complete questionnaires (as described below).

At T_0_ and T_1_ participants visited the DCCN in Nijmegen for the following study aspects and assessments:

- **Signing informed consent forms**. At T_0_ the full written informed consent form was signed in attendance of an involved research team member. Additionally, an MRI-screening and informed consent form was signed to check MRI-safety issues before actual scanning commences, both at T_0_ and T_1_.

- **Information and practicing task**. Participants received information about the course of the day and practice the working memory n-back task.

- **MRI-scanning**. Participants underwent MRI-scanning of the brain and abdomen. Only after participants had been deemed metal free and safe to enter the MRI-room, the scan took place. The preparation and scans together had a duration of approximately 1.5 hours.

- **Anthropometrics and blood pressure assessment**. Length and weight were measured before MRI scanning. Blood pressure and heartrate were measured twice (or three times in case of large differences between first two measurements) on the non-dominant arm using an electronic arm blood pressure monitor (Omron X3). Participants had no coffee or tea before blood pressure were measured, to avoid influences of caffeine. Hip- and waist circumference were measured using a tape measure.

- **Transit time analysis**. Participants were asked to eat two muffins containing blue colouring agents (E133, E122) within 10 minutes, in accordance with the ‘blue poo’ method [5]. Participants received an information leaflet and gastrointestinal questionnaires to fill in at home.

- **Neuropsychological test battery (NTB).** Participants underwent a cognitive assessment for executive function, working memory and processing speed. The full assessment was performed in a neutral room and took approximately 45 minutes.

- **Exchange of materials and information**. Participants received the materials required to collect and store three faecal samples in their freezer, ingredients for a standardized meal to be consumed on the evening before the WUR study centre visit, a Chill+ smartwatch provided by IMEC Oneplanet (Wageningen, the Netherlands), and corresponding instructions. The smartwatch measured skin temperature, galvanic skin response, and movement. Participants were asked to wear this smart-watch for 7 consecutive days (1 week) during day and night and were instructed to charge the Chill+-smartwatch for at least an hour every day, preferably on the same time point. Additionally, participants received an invitation by e-mail via Castor EDC and Eetscore to fill in lifestyle domain-related questionnaires. Participants could fill in these questionnaires at their own pace at home.

In total, the DCCN study centre visit took approximately 4 hours from start to finish.

After this week, at T_0_ and T_1_, participants visited the WUR whilst fasted since 20:00 the evening before, for the following study aspects and assessments:

- **Collect faecal samples**. In addition, the smartwatch and completed questionnaires were returned.

- **Blood drawing**. Blood was drawn via a finger-prick for direct measurement of CRP and white blood cell counts, and intravenously from the arm. In total, 25 mL blood was collected: 6 mL in a serum tube, 6 mL in a lithium heparin plasma tube, 2 mL in a natrium fluoride plasma tube, and 13 mL in two K_2_EDTA tubes, of which 3 mL for whole blood collection and analyses, and the 10 mL for plasma collection.

- **Urine collection**. Urine was collected during the visit on location.

- **SIBO detection**. A hydrogen breath-analysis after consumption of 75 gram glucose was performed to measure potential presence of SIBO. Diabetes patients were exempted from the SIBO breath test.

In total, the WUR study centre visit took approximately 3 hours from start to finish.

In Supplementary Table 2 below, a summary of the complete participant timeline can be found, including enrollment, assessments, outcomes and interventions.

**Table S2. Schedule of enrollment, interventions, assessments and outcomes**

| **Study phase** | **Enrollment** | **Baseline visits** | **Randomization** | **Intervention period** | | | | | | | **Follow-up visits** |
| --- | --- | --- | --- | --- | --- | --- | --- | --- | --- | --- | --- |
| **Duration per wave** | Continuous | 2-3 months | 1 week | 6 months (26 weeks) | | | | | | | 1-1.5 months |
| **Time point** | E | **T_0_** | R | m_1_ | m_2_ | m_3_ | **T_1/2_** | m_4_ | m_5_ | m_6_ | **T_1_** |
|  | | | | | | | | | | | |
| **Enrollment** | | | | | | | | | | | |
| Registration via online form | X |  |  |  |  |  |  |  |  |  |  |
| First phone call | X |  |  |  |  |  |  |  |  |  |  |
| Screening informed consent | X |  |  |  |  |  |  |  |  |  |  |
| Telephonic screening (TICS) | X |  |  |  |  |  |  |  |  |  |  |
| Informed consent |  | X |  |  |  |  |  |  |  |  |  |
| Allocation |  |  | X |  |  |  |  |  |  |  |  |
| **Interventions** | | | | | | | | | | | |
| High-intensity multidomain lifestyle intervention |  |  |  |  |  |  |  |  |  |  |  |
| Low-intensity multidomain lifestyle intervention |  |  |  |  |  |  |  |  |  |  |  |
| **Assessments** | | | | | | | | | | | |
| Neuroimaging |  | X |  |  |  |  |  |  |  |  | X |
| Neuropsychology |  | X |  |  |  |  |  |  |  |  | X |
| Anthropometry |  | X |  |  |  |  |  |  |  |  | X |
| Faeces (3x) |  | X |  |  |  |  |  |  |  |  | X |
| Blood |  | X |  |  |  |  |  |  |  |  | X |
| Urine |  | X |  |  |  |  |  |  |  |  | X |
| Breath |  | X |  |  |  |  |  |  |  |  | X |
| Questionnaires |  | X |  | X | X | X | X | X | X | X | X |
| Smartwatch |  | X |  |  |  |  |  |  |  |  | X |
| **Outcomes** | | | | | | | | | | | |
| *Primary* | | | | | | | | | | | |
| dlPFC and hippocampus brain activity during working memory |  | X |  |  |  |  |  |  |  |  | X |
| Working memory performance |  | X |  |  |  |  |  |  |  |  | X |
| dlPFC and hippocampus cerebral perfusion levels |  | X |  |  |  |  |  |  |  |  | X |
| Blood IL-6, TNF-α, hs-CRP levels |  | X |  |  |  |  |  |  |  |  | X |
| Faecal microbiota diversity |  | X |  |  |  |  |  |  |  |  | X |
| *Secondary* | | | | | | | | | | | |
| Body mass index |  | X |  |  |  |  |  |  |  |  | X |
| Waist circumference |  | X |  |  |  |  |  |  |  |  | X |
| Hip circumference |  | X |  |  |  |  |  |  |  |  | X |
| Blood pressure |  | X |  |  |  |  |  |  |  |  | X |
| Abdominal fat distribution |  | X |  |  |  |  |  |  |  |  | X |
| Brain structure volume profile |  | X |  |  |  |  |  |  |  |  | X |
| Whole brain activity during working memory |  | X |  |  |  |  |  |  |  |  | X |
| Whole brain cerebral perfusion levels |  | X |  |  |  |  |  |  |  |  | X |
| Brain neuroinflammation levels |  | X |  |  |  |  |  |  |  |  | X |
| Neuropsychological test battery scoring |  | X |  |  |  |  |  |  |  |  | X |
| Lifestyle domain adherence questionnaires |  | X |  | HI | HI | HI | X | HI | HI | HI | X |
| Smartwatch-based lifestyle adherence measures |  | X |  |  |  |  |  |  |  |  | X |
| Faecal microbiota composition |  | X |  |  |  |  |  |  |  |  | X |
| Short-chain fatty acids (SCFAs) profile in faeces |  | X |  |  |  |  |  |  |  |  | X |
| Metabolomics in faeces |  | X |  |  |  |  |  |  |  |  | X |
| Intestinal inflammation markers in faeces |  | X |  |  |  |  |  |  |  |  | X |
| Gut transit time |  | X |  |  |  |  |  |  |  |  | X |
| Metabolic profile in blood |  | X |  |  |  |  |  |  |  |  | X |
| Inflammation profile in blood |  | X |  |  |  |  |  |  |  |  | X |
| Intestinal integrity profile in blood |  | X |  |  |  |  |  |  |  |  | X |
| Brain health profile in blood |  | X |  |  |  |  |  |  |  |  | X |
| White blood cell count in blood |  | X |  |  |  |  |  |  |  |  | X |
| Small intestinal bacterial overgrowth |  | X |  |  |  |  |  |  |  |  | X |
| *Other* | | | | | | | | | | | |
| Baseline demographics questionnaire |  | X |  |  |  |  |  |  |  |  |  |
| Intracranial iron deposition and myelin |  | X |  |  |  |  |  |  |  |  | X |
| Urine microbiota-derived bioactive compounds |  | X |  |  |  |  |  |  |  |  | X |
| Gastro-intestinal questionnaires |  | X |  |  |  |  |  |  |  |  | X |
| Other questionnaires |  | X |  |  |  |  |  |  |  |  | X |

Abbreviations: *dlPFC* dorsolateral prefrontal cortex*; HI* supervised High-Intensity coaching intervention group; *hs-CRP* high-sensitivity C-reactive protein; *IL-6* interleukin-6; *LI* unsupervised Low-Intensity coaching intervention group*; TICS* Telephone Interview for Cognitive Status; *TNF-α* tumor necrosis factor alpha

**Table S3. HELI MRI sequence protocol.**

| **Scan type** | **Sequence** | **TR**  **TI1/TI2**  **(ms)** | **TE**  **(ms)** | **Voxel size (mm)** | **Flip angle (°)** | **FOV**  **(AP/RL/FH; mm)** | **Acceleration** | **Acquisition Time (min:sec)** | **Comments/other** |
| --- | --- | --- | --- | --- | --- | --- | --- | --- | --- |
| **T1-weighted** | MP2RAGE | 6000  700/2400 | 2.34 | 1.0 × 1.0 × 1.0 | 6/6 | 216/176/256  (sagittal slab positioning) | iPAT 3 | 7:32 | Used for anatomical reference and R1 maps |
|  | VIBE | 7 | TE_1_/TE_2_ =  2.46/3.69 | 1.2 × 1.2 × 3.0 | 9 | 274/380/108  (perpendicular between L4/L5 spinal segments) | CAIPI 6  (2y,3z,2 shiftz) | 0:10 | Abdominal T1 for visceral/subcutaneous adiposity distribution |
| **B1 map** | turboFLASH | 10000 | 2.23 | 3.3 × 3.3 × 2.5 (2.5 slice spacing) | Sat: 80  Exc: 8 | 212/212/210 | - | 0:20 | Used for B1 |
| **MRS** | PRESS | 2000 | 35 | 20 × 20 × 20 | 90x180x180 | 20/20/20 (in dlPFC and hippocampus) | - | 2:18  (64 averages) | With water suppression |
|  | PRESS | 2000 | 35 | 20 × 20 × 20 | 90x180x180 | 20/20/20  (in dlPFC and hippocampus) | - | 0:42  (16 averages) | No water suppression, for reference |
| **WM task-based fMRI** | EPI | 1500 | 33.40 | 2.0 × 2.0 × 2.0 | 75 | 213/213/136  (transversal slab positioning) | Multi band 4 | 16:26 | N-back (0b, 1b, 2b) working memory task |
| **Perfusion imaging** | ASL | 4000  Saturation mode 90  And WET background Suppression | 16.28 | 1.8 × 1.8 × 3.5 | Exc: 90  Ref: 120 | 224/146/80  (transversal slab positioning) | TurboFactor12  EPI Factor 21  CAIPI 4  (2y,2z,1 shiftz) XA platform | 4:20 | SubBolus Dur: 400ms  RF FA 25 RFgap = PLD 100ms  8-PLD time-encoded pCASL |
|  | M0 | 3000  Saturation mode 90 | 16.28 | 1.8 × 1.8 × 3.5 | 120 | 224/146/80  (transversal slab positioning) | TurboFactor12  EPIFactr 21  CAIPI 4  (2y,2z,1 shiftz) XA platform | 0:39 | Calibration image for time-encoded pCASL (500ms /1500ms /2500ms) |
| **R2*/ QSM** | Multi-echo GRE | 40 | TE_1_/ΔTE/TE_9_ =  3.54/5.32/35.46 | 0.9 × 0.9 × 0.9 | 15 | 230/188/266  (sagittal slab positioning) | CAIPI6  (1y,6z,2 shiftz) | 8:34 | Magnitude and phase saved, used for R2* and QSM computation |

*Abbreviations: AP* anterior-posterior; *ASL* arterial spin labelling; *CAIPI* controlled aliasing in parallel imaging; *dlPFC* dorsolateral prefrontal cortex; *EPI* echo planar imaging; *FH* foot-head; *fMRI* functional magnetic resonance imaging; *FOV* field of view; *GRE* Gradient Echo Imaging; *iPAT* integrated parallel acquisition technique; *M0* equilibrium magnetization; *MP2RAGE* Magnetization Prepared 2 Rapid Acquisition Gradient Echoes; *MRS* magnetic resonance spectroscopy; *pCASL* pseudocontinuous arterial spin labelling; *PLD* post-labelling delay; *PRESS* Point REsolved Spectroscopy; *QSM* quantitative susceptibility mapping; *RF FA* radiofrequency flip angle; *RL* right-left; *TE* echo time; *TI* inversion time; *TR* repetition time; *VIBE* Volumetric Interpolated Breath-hold Examination; *WM* working memory

# S5. Sample size

| **Table S4.** Overview of effects and effect sizes of previous relevant lifestyle (intervention) studies | | | | | | |
| --- | --- | --- | --- | --- | --- | --- |
| **Paper** | | **Lifestyle domain(s)** | **Intervention duration** | **Active control** | **Observed effect(s)** | **Effect size** |
|  | |  |  |  | **Brain function**  **(MRI & cognition)** |  |
| Kleinloog *et al. [6]*  Frontiers aging Neuroscience  Dec. 2019 | | Exercise | 2 months  3x 50 min per week | × | ↑frontal lobe CBF | 0.393 – 0.552  (*d*) |
| Thomas *et al. [7]*  J. Alzheimer’s Disease: JAD  May. 2020 | | Exercise | 1 year  3x 30 min per week | ✔ | ↑hippocampal CBF | 0.58 (*d*) |
| Li *et al. [8]*  Frontiers Aging Neuroscience  Mar. 2014 | | Exercise  and  Cognition  (+Social) | 6 weeks  3x 1h cognitive and exercise group sessions per week | ✔ | ↑RS functional connectivity between medial PFC and medial temporal lobe | -0.036 to 0.201  (*z(r))* |
| Valls-Pedret *et al*. [9]  J. Alzheimer’s Disease: JAD  Jan. 2012 | | Diet | 1 year  Weekly meal plan | ✔ | ↑(working) memory & MMSE in Med. diet components | 0.094 – 0.149  (*β*) |
| Hardman *et al*. [10]  Curr. Dev. in Nutrition: CDN  Apr. 2020 | | Diet  and/or  Exercise | 6 months  Increasing walking times |  | ↑Working memory (only in combined training) | 0.06 (*η^2^*) |
| Oswald *et al*. [11]  European Journal of Ageing  Nov. 2006 | | Cognition  and/or  Exercise | 30 weeks  1x 45 min cognition or 90 min cognition and exercise per week | ✔ | ↑composite cognitive function (in combined training) | 0.75 (*d*) |
| Joubert & Chainay [12]  Clinical Interv. in Aging  Jul. 2018 | | Cognition  and/or Exercise | N/A:  Systematic review | × | Both cognitive and physical training preferentially impact and enhance specific brain structures and function | Globally:  *η^2^* 0.001 – 0.88 in cognitive training;  0.01 – 0.97 in physical training;  0.14 – 0.34 in combined |
|  | |  |  |  | **Inflammation** |  |
| Ruangthai *et al*. [13]  Arch. Of Gerontol. and Geriatr.  Oct. 2020 | | Exercise | 3 months  3x 1 hr combined land or combined water- based exercise | × | ↓hs-CRP levels in both combined land or combined water-based exercise compared to control | 0.7 – 1.1  (ES given as a standardized calculation of differences in group means*) |
| Sanada *et al*. [14]  Int. J. of Molecular Sciences  Apr. 2020 | | Mindfulness | N/A  Systematic  Review & meta-analysis | × | ↓IL-6, TNF-α, and hsCRP after mindfulness-based intervention | 0.6 – 0.66 (*d*) |
| Corrêa *et al*. [15]  Scientific Reports  Jul. 2020 | | Exercise | 3 months  3x 50 min weekly resistance training | × | ↓TNF-α levels, also associated with increased total sleep time affected by exercise | -0.23 (*r*) |
|  | |  |  |  | **Microbiome** |  |
| Grosicki *et al*. [16]  Sleep Medicine  Sep. 2020 | | Sleep | 1 month  Self-reported sleep quality | × | ↑Gut microbial diversity in high self-reported sleep quality | -0.394 – -0.566 (*r*)  and  0.474 – 0.553 (*r*) |
| Li *et al*. [17]  Nature and Science of Sleep  Nov. 2020 | | Sleep | 1 month  self-reported sleep quality assessment | × | ↑microbiome richness and diversity associated with superior sleep quality | 0.631 – 0.721 (*r*) |
| Morita *et al*. [18]  Nutrients  Apr. 2019 | | Exercise | 3 month  Weekly 1h group training | ✔ | ↑microbiome (*Bacteroides*) correlation following exercise | 0.371 – 0.431 (*r*) |
|  | |  |  |  | **Microbiome-cognition links** |  |
| Bagga *et al*. [19]  Gut Microbes  Nov. 2018 | | Dietary supplement | 1 month use of multispecies probiotics | ✔ | ↑emotional memory, correlated with microbiome (*Bacteroides*) | 0.3 – 0.4 (*r*) |
| Bloemendaal *et al*. [20]  Translational Psychiatry  Apr. 2021 | | Dietary supplement | 1 month use of multispecies probiotics | ✔ | ↑stress-related working memory performance, correlated with (*Ruminococcaceae UCG003*) | 0.565 (*r*) |
| *r*  *d*  *η^2^*  * | 0.1 (small), 0.3 (medium), 0.5 (large)  0.2 (small), 0.5 (medium), 0.8 (large)  ≥ 0.01 (small), ≥ 0.06 (medium), ≥ 0.14 (large)  The standardized differences in means ES of 0.6 – 1.1 approximately translates to a Cohen’s *d* of 0.3 – 0.5 | | | | | |

# S6. Results

| **Table S5.** Baseline characteristic comparison of HELI study with previous multidomain lifestyle intervention studies | | | | |
| --- | --- | --- | --- | --- |
| **Study reference** | **Age (years)** | **Sex (females)** | **Education** | **Risk factors used for inclusion criteria and comparison of reported lifestyle-related risks** |
| HELI study  (present study) | 66.6 (± 4.3) | 67/102 (65.7%) | 16.2 (± 4.6) total years  10/102 (9.8%) low level (ISCED 0-2)  37/102 (36.3%) medium level (ISCED 3-4)  55/102 (53.9%) high level (ISCED 5-8) | Inclusion based on 2 or more lifestyle modifiable risk factors of CAIDE dementia risk score:  Overweight: 76/102 (74.5%)  Physical inactivity: 57/102 (55.9%)  Hypertension: 58/102 (56.9%) Hypercholesterolemia: 57/102 (55.9%)  Diabetes: 20/102 (19.6%)  Mild cardiovascular disease: 11/102 (10.8%) |
| FINGER-study  Kivipelto *et al*. [21] | 69.3 (± 4.7) | 551/1190 (46.4%) | 10.0 (± 3.4) total years | Inclusion based on CAIDE dementia risk score of 6 or higher.  Physical inactivity: 343/1180 (29.1%)  Hypertension: 779/1177 (66.2%) Hypercholesterolemia: 803/1180 (68.1%)  Diabetes: 150/1180 (12.7%) |
| Pre-DIVA  Richard *et al*. [22] | 74.4 (± 2.3) | 552/1004 (55.0%) | 271/1004 (27%) ≤7 total years  615/1004 (61.3%) 7-12 total years  117/1004 (11.7%) ≥12 total years | Inclusion based on age and previous admission to general practitioner.  Overweight: 743/1004 (74.0%)  Physical inactivity: 94/1004 (9.4%)  Mean SBP ± SD: 157 (21)  Mean DBP ± SD: 80 (11)  Total cholesterol >6.5mmol/L: 224/1004 (22.3%)  Diabetes: 204/1004 (20.3%) |
| MAPT study  Vellas *et al*. [23] | 75.3 (± 4.4) | 1088/1680 (64.8%) | 85/1680 (5.2%) no diploma  286/1680 (17.4%) primary school certificate  553/1680 (33.7%) secondary education without high-school diploma  719/1680 (43.8%) high-school diploma or higher | Inclusion based on age (≥70 years) with spontaneous memory complaints, limitation in one instrumental activity of daily living, and slow gait.  Mean BMI ± SD: 26.1 (4.1)  Mean SBP ± SD: 141.0 (19.7)  Mean DBP ± SD: 78.8 (11.5) |
| J-MINT  Sakurai *et al*. [24] | 74.5 (± 4.9) | 226/433 (52.2%) | 12.6 (± 2.5) total years | Inclusion based age (65-85 years) with cognitive decline adjusted for age and education.  No lifestyle-related risk factors reported. |
| AgeWell.de trial  Zülke *et al. [25]* | 69.0 (± 4.9) | 611/1176 (52.0%) | 13/1030 (1.3%) ≤7 total years  456/1030 (44.3%) 7-9 total years  561/1030 (54.5%) ≥10 total years  227/1030 (22.1%) CASMIN 1 (i.e. ISCED 0-2)  546/1030 (53.0%) CASMIN 2 (i.e. ISCED 3-4)  256/1030 (24.9%) CASMIN 3 (i.e. ISCED 5-8) | Inclusion based on age (60-77 years) and a CAIDE dementia risk score of 9 or higher.  Obese: 676/1176 (57.5%)  Physical inactivity: 301/1176 (25.6%)  Hypertension: 1000/1176 (85.0%)  Hypercholesterolemia: 633/1176 (53.8%) |
| *Abbreviations: BMI* Body Mass Index; *CAIDE* Cardiovascular Risk Factors, Aging and Incidence of Dementia [26]; *CASMIN* Comparative Analysis of Social Mobility in Industrial Nations [27]; *DBP* diastolic blood pressure; *ISCED* International Standard Classification of Education [28]; *SBP s*ystolic blood pressure; SD standard deviation;  Values are described as mean (±SD), or as *n*/total number of participants (%). The column with the comparison of lifestyle-related risks only shows data of lifestyle-related risk which has also been collected by the HELI study. | | | | |

# S7. Data

## S7.1 Data collection plan

Researchers were trained properly before they were allowed to perform screenings and measurements independently (e.g. TICS, neuropsychological testing, anthropometrics, breathing test). The neuroimaging assessments were performed by MRI-certified researchers only. Blood was collected by nurses and directly processed by trained lab technicians. If possible, measurements (e.g. blood pressure, heart rate) were performed in duplicate.

## S7.2 Data management

Castor EDC was used as an electronic case report form (CRF) and allowed for careful monitoring data entrance. At the DCCN, a paper version of the CRF was used during the test session, and test session information was filled in on Castor EDC directly afterwards. At the WUR, test session information was filled in directly on Castor EDC, without use of a paper version of the CRF.

Neuroimaging data collected at the DCCN was directly transferred and stored securely on the Radboud Data Repository (RDR; [https://data.ru.nl](https://data.ru.nl/)). Processing of all data on computers is protected by a site firewall. Restricted access to study data on the central storage system is implemented by means of specific user groups. Participant metadata is stored in password-protected databases. Details about data management procedures can be found on the website of the RDR.

Blood marker, microbiota composition and metabolite data analysed at the WUR is safely stored, archived, and registered in the Wageningen University data storage system according to the regulations of the WUR, and implemented within the Division of Human Nutrition and Health.

# S8. Monitoring

## S8.1 Data monitoring: formal committee (21a)

This study has a low risk for participants, and the participants are of a low-risk group. Therefore, a data monitoring committee (DMC) is not appointed for this study.

## S8.2 Data monitoring: interim analysis (21b)

All research conducted at the DCCN meets with the assessment of negligible-minimal risk- assessment as defined in the Dutch Federation Academic Medical Centers (NFU) indicating onsite minimal monitoring. The Clinical Research Coordinator is assigned as designated on-site monitor. The CRC/monitor will assess the HELI study at least once during the data acquisition phase. Outcomes will be documented and reported to the principal investigator. In case of a critical finding the director will be notified by the CRC. He may decide to put the study on hold. Resuming the study requires the director’s formal approval. Besides the annual reporting of monitor visits the Donders Management Team will also be notified of the occurrences of serious protocol breaches (violations). In all cases of study termination, appropriate steps will be taken to ensure the safety of study participants.

A study monitoring interim visit was conducted to ensure that the investigator and site were compliant with the study protocol, that data will be of high quality and integrity and that the facilities and staffing were adequate to continue best practices. The monitoring procedures on site concern all investigator-driven interventional research conducted at the DCCN.

The monitoring standard operating procedure (SOP) applies to all site personnel involved in the implementation, coordination and conduct of investigator-initiated interventional research. The principal investigator scheduled and arranged the monitoring visits as requested by the monitor. A monitor visit consisted of an occasional study sample taken at random during the initiation phase of the study. Monitoring visits were scheduled at mutually convenient times and every attempt was made to accommodate the monitoring visit. Prior to each visit, the principal investigator designee confirmed with the monitor what materials were to be reviewed so that appropriate documentation and files were made readily available. These materials included:

• All available study-specific Informed Consent forms;

• Subject source documents and corresponding case report forms (CRFs) and/or access to the data management tool CastorEDC;

• Safety reports and/or Adverse event documentation;

• Study Site Master File/ Regulatory Binder;

• Applicable SOP’s.

## S8.3 Harms (22)

Adverse events (AE) are defined as any undesirable experience occurring to a subject during the study, whether or not considered related to (the investigational product / trial procedure/ the experimental intervention). All AEs reported spontaneously by the subject or observed by the investigator or his staff will be recorded. A serious adverse event (SAE) is any untoward medical occurrence or effect that:

Results in death;

Is life threatening (at the time of the event);

Requires hospitalization or prolongation of existing inpatients’ hospitalization;

Results in persistent or significant disability or incapacity;

Is a congenital anomaly or birth defect; or

Any other important medical event that did not result in any of the outcomes listed above due to medical or surgical intervention but could have been based upon appropriate judgement by the investigator.

An elective hospital admission will not be considered as a serious adverse event.

The staff will report all SAEs to the principal investigator as soon as possible after they have been observed. The investigator will report these to the sponsor without undue delay after obtaining knowledge of the events. The sponsor will report the SAEs to the accredited METC that approved the protocol, within 7 days of first knowledge for SAEs that result in death or are life threatening followed by a period of maximum of 8 days to complete the initial preliminary report. All other SAEs will be reported within a period of maximum 15 days after the sponsor has first knowledge of the serious adverse events.

## S8.4 Auditing (23)

Apart from a standard monitor initiation, interim and closing-out visits, no specific scheduled auditing visits are planned for this study.

# S9. Ethics and dissemination

## S9.1 Research ethics approval (24)

This research study falls under the Medical Research Involving Human Subjects Act (WMO). The protocol has been reviewed and approved by the MREC Oost-Nederland of the Central Committee on Research Involving Human Subjects (CCMO), under approval number NL78263.091.21.

## S9.2 Protocol amendments (25)

All substantial amendments will be notified to the METC that gave a favorable opinion. A ‘substantial amendment’ is defined as an amendment to the terms of the METC application, or to the protocol or any other supporting documentation, that is likely to affect to a significant degree:

- The safety or physical or mental integrity of the subjects of the trial;
- The scientific value of the trial;
- The conduct or management of the trial; or
- The quality or safety of any intervention used in the trial.

Non-substantial amendments will not be notified to the accredited METC and the competent authority, but will be recorded and filed by the sponsor.

## S9.3 Consent or assent (26a)

Written informed consent are only signed and collected by research team members directly involved with the present study.

## S9.4 Confidentiality (27)

Handling of personal data will comply EU privacy law, the General Data Protection Regulation (GDPR) and the Dutch Act on Implementation (in Dutch: Uitvoeringswet AVG, UAVG).

The Donders Institute has established a data management infrastructure ([https://data.donders.ru.nl](https://data.donders.ru.nl/)) to which all relevant data will be stored online in a central storage space during data collection. Research data will be copied to a central storage system. Processing of all data on computers is protected by a site firewall. Restricted access to study data on the central storage system is implemented by means of specific Unix user groups. Participant metadata is stored in password-protected databases. A second copy of the data is stored on the Radboud Data Repository (RDR) located on the Radboud University campus. Raw data will be archived according to institutional standards. For data processing, raw data will be uploaded to network attached storage that, in compliance with institutional guidelines, only researchers directly involved in the processing of data from the specific project will be able to access.

The investigator will ensure that the subject's anonymity will be maintained. In all documents, subjects will be identified by an identification code only – i.e., not by their names or any other feature by which subjects can be identified. The investigator will keep a separate Subject Identification Code List (Key-file), which matches identifying codes with the subjects’ names. Key-files will be maintained by the investigator in a strict confidential way and preserved for a certain period of time meeting with demonstrable legitimate interest (according to GDPR). Subject anonymity will be maintained throughout all archiving steps. The identification code is shared with our multicenter partner WUR, so the data from the gathered at the Donders Institute and at Wageningen University is placed under the same identification code of an individual.

## S9.5 Declaration of interests (28)

This work was supported by a Crossover grant (MOCIA 17611) of the Dutch Research Council (NWO). The MOCIA programme is a public-private partnership (see <https://mocia.nl/scientific/>). There are no competing interests to be declared.

## S9.6 Data access (29)

Access to data is managed by the researchers involved with the study and responsible for the data, and only directly involved research team members will be able to give access rights to other individuals when necessary or requested.

## S9.7 Ancillary and post-trial care (30)

The sponsor/investigator has a liability insurance which is in accordance with article 7 of the WMO. The sponsor (also) has an insurance which is in accordance with the legal requirements in the Netherlands (Article 7 WMO). This insurance provides cover for damage to research subjects through injury or death caused by the study. The insurance applies to the damage that becomes apparent during the study or within 4 years after the end of the study.

## S9.8 Dissemination policy: trial results (31a)

Trial results will be published in open-access peer-reviewed journals.

## S9.10 Dissemination policy: authorship (31b)

Authorship eligibility guidelines and any intended use of professional writers

Authorshop guidelines are compliant with the Radboud University (Medical Center) Guidelines for publication and authorship. These guidelines are intended to provide a description of the conditions regarding eligibility for authorship, and to determine which order of authorship is the most appropriate.

*Conditions for authorship*

a. Each author must have made a substantial contribution to the creative concept, the design or execution of the research described in the article and/or to the analysis or interpretation of the data.

b. Each author must have made a substantial contribution to the drawing up or critical assessment of at least part of the intellectual concepts in the article.

c. Without prejudice to the specific responsibility for his/her own contribution, each author should have given his/her approval for the final version of the entire manuscript.

d. One of the authors must take responsibility for the article in its entirety (in principle the author to which any related correspondence is to be addressed).

e. Neither the routine provision of data or material nor the facilitation of financial resources justifies co-authorship. A reference in the acknowledgements section to the contribution in question will usually suffice.

*Basic principles for determining order of authorship*

a. Where possible, agreements regarding authorship should be made beforehand.

b. The first author is the researcher who has made the greatest contribution to the project, including its execution: for example, a PhD student or postdoctoral fellow. The second author is the author that has made the second most important contribution.

c. The last author has developed the key creative concept of the research and has supervised the research. The last author is generally the research group leader. In this respect, the penultimate author has played the second most important role. The head of department is not by default the last author because of his/her position.

d. A shared first or last authorship is only possible in the event of equal contributions.

## S9.11 Dissemination policy: reproducible research (31c)

Papers will only be submitted to, and published in, open-access journals. The data used will be shared on a Data Sharing Collection of the RDR of the Radboud University, Nijmegen, The Netherlands.

# S10. Informed consents

## S10.1 Informed consent HELI study: English version

Belonging to the HELI study.

- I have read the information letter. I was able to ask questions. My questions have been answered well enough. I had enough time to decide if I want to participate.
- I know that participating is voluntary. I also know that I can decide not to take part in the study at any time, or to stop participating. I do not have to explain why.
- I am willing to participate in all elements of this study which have been described in this information letter (such as visiting the research centers, partaking in the lifestyle intervention).
- I consent to the researcher to inform my general practitioner about my participation in this study.
- I consent to the researcher to disclose information to my general practitioner about possible incidental findings from this study which could have a clinical significance for my health.
- I consent to the collection, use and storage of my data and biomaterial. The researchers will only use this data to answer the research question of this study as explained in the information letter.
- I consent to the storage of my data and biomaterial, for a minimal period of 15 years.
- I am aware that for the purpose of monitoring this research study some designated people are able to look into my data. These designated people have been mentioned in the information letter. I consent to these designated people to access my data for monitoring purposes.
- Would you please tick ‘Yes’ or ‘No’ in the table below?

| I consent to my data and biomaterial to be stored, and to be possibly used for other research, as mentioned in the information letter. | Yes ☐ | No ☐ |
| --- | --- | --- |
| I consent to my (leftover) data and biomaterial to be stored, and to be possibly used for other future research, as mentioned in the information in the information letter. The biomaterial will be stored for this purpose for a minimum of 15 years. | Yes ☐ | No ☐ |
| I consent to be approached after this study to participate in future studies. | Yes ☐ | No ☐ |
| I consent to the researchers informing me which treatment I received/which group I was in. | Yes ☐ | No ☐ |

- I want to participate in this research study.

My name is (participant): ………………………………..

Signature: ……………………… Date: __ / __ / __

-----------------------------------------------------------------------------------------------------------------

*To be filled in by the researcher:*

I declare that I have fully informed this participant with regard to the said research study.

Will information become known during the research study which could impact or influence the consent of the participant? Then I will inform the participant in time.

Name researcher (or his/her representative):……………………………….

Signature:……………………… Date: __ / __ / __

-----------------------------------------------------------------------------------------------------------------

*The participant receives a complete information letter, together with a signed version of the informed consent form.*

## S10.2 Informed consent HELI study: Dutch version (original)

Behorende bij de HELI studie.

- Ik heb de informatiebrief gelezen. Ook kon ik vragen stellen. Mijn vragen zijn goed genoeg beantwoord. Ik had genoeg tijd om te beslissen of ik meedoe.
- Ik weet dat meedoen vrijwillig is. Ook weet ik dat ik op ieder moment kan beslissen om toch niet mee te doen met het onderzoek. Of om ermee te stoppen. Ik hoef dan niet te zeggen waarom ik wil stoppen.
- Ik ben bereid mee te doen aan alle onderdelen van dit onderzoek die in deze informatiebrief beschreven staan (o.a. de bezoeken aan onderzoekscentra, leefstijlinterventie).
- Ik geef de onderzoeker toestemming om mijn huisarts te laten weten dat ik meedoe aan dit onderzoek.
- Ik geef de onderzoeker toestemming om mijn huisarts informatie te geven over onverwachte bevindingen uit het onderzoek die van belang zijn voor mijn gezondheid.
- Ik geef de onderzoekers toestemming om mijn gegevens en lichaamsmateriaal te verzamelen en gebruiken. De onderzoekers doen dit alleen om de onderzoeksvraag van dit onderzoek te beantwoorden.
- Ik geef de onderzoekers toestemming dat mijn gegevens 15 jaar bewaard mogen worden op de onderzoekscentra.
- Ik weet dat voor de controle van het onderzoek sommige mensen al mijn gegevens kunnen inzien. Die mensen staan in deze informatiebrief. Ik geef deze mensen toestemming om mijn gegevens in te zien voor deze controle.
- Wilt u in de tabel hieronder ja of nee aankruisen?

| Ik geef toestemming om mijn gegevens te bewaren om dit te gebruiken voor ander onderzoek, zoals in de informatiebrief staat. | Ja ☐ | Nee☐ |
| --- | --- | --- |
| Ik geef toestemming om mijn (overgebleven) lichaamsmateriaal te bewaren om dit te gebruiken voor ander toekomstig onderzoek, zoals in de informatiebrief staat. Het lichaamsmateriaal wordt daarvoor nog minimaal 15 jaar bewaard. | Ja ☐ | Nee☐ |
| Ik geef toestemming om mij eventueel te vragen of ik wil meedoen met ander onderzoek. | Ja ☐ | Nee☐ |
| Ik geef de onderzoekers toestemming om na het onderzoek te laten weten welke behandeling ik heb gehad/ in welke groep ik zat. | Ja ☐ | Nee☐ |

- Ik wil meedoen aan dit onderzoek.

Mijn naam is (proefpersoon): ………………………………..

Handtekening: ……………………… Datum : __ / __ / __

-----------------------------------------------------------------------------------------------------------------

*In te vullen door de onderzoeker:*

Ik verklaar dat ik deze proefpersoon volledig heb geïnformeerd over het genoemde onderzoek.

Wordt er tijdens het onderzoek informatie bekend die die de toestemming van de proefpersoon kan beïnvloeden? Dan laat ik dit op tijd weten aan deze proefpersoon.

Naam onderzoeker (of diens vertegenwoordiger):……………………………….

Handtekening:……………………… Datum: __ / __ / __

-----------------------------------------------------------------------------------------------------------------

*De proefpersoon krijgt een volledige informatiebrief mee, samen met een getekende versie van het toestemmingsformulier.*

# S11. References

113. van den Berg, E., et al., *The Telephone Interview for Cognitive Status (Modified): relation with a comprehensive neuropsychological assessment.* J Clin Exp Neuropsychol, 2012. **34**(6): p. 598-605.

120. van Emmerik, A.A.P., F. Berings, and J. Lancee, *Efficacy of a Mindfulness-Based Mobile Application: a Randomized Waiting-List Controlled Trial.* Mindfulness (N Y), 2018. **9**(1): p. 187-198.

121. Reijnders, J.S.A.M., et al., *“Keep your brain fit!” Effectiveness of a psychoeducational intervention on cognitive functioning in healthy adults: A randomised controlled trial.* Neuropsychological Rehabilitation, 2017. **27**(4): p. 455-471.

122. Van der Zweerde, T., et al., *Nurse-Guided Internet-Delivered Cognitive Behavioral Therapy for Insomnia in General Practice: Results from a Pragmatic Randomized Clinical Trial.* Psychother Psychosom, 2020. **89**(3): p. 174-184.

140. Asnicar, F., et al., *Blue poo: impact of gut transit time on the gut microbiome using a novel marker.* Gut, 2021.

52. Kleinloog, J.P.D., et al., *Aerobic Exercise Training Improves Cerebral Blood Flow and Executive Function: A Randomized, Controlled Cross-Over Trial in Sedentary Older Men.* Front Aging Neurosci, 2019. **11**: p. 333.

53. Thomas, B.P., et al., *Brain Perfusion Change in Patients with Mild Cognitive Impairment After 12 Months of Aerobic Exercise Training.* J Alzheimers Dis, 2020. **75**(2): p. 617-631.

107. Ruangthai, R., et al., *Comparative effects of water- and land-based combined exercise training in hypertensive older adults.* Archives of Gerontology and Geriatrics, 2020. **90**.

14. 106. Corrêa, H.L., et al., *Resistance training improves sleep quality, redox balance and inflammatory profile in maintenance hemodialysis patients: a randomized controlled trial.* Scientific Reports, 2020. **10**(1): p. 11708.

99. Grosicki, G.J., et al., *Self-reported sleep quality is associated with gut microbiome composition in young, healthy individuals: a pilot study.* Sleep Medicine, 2020. **73**: p. 76-81.

95. Morita, E., et al., *Aerobic Exercise Training with Brisk Walking Increases Intestinal Bacteroides in Healthy Elderly Women.* Nutrients, 2019. **11**(4).

153. Bagga, D., et al., *Probiotics drive gut microbiome triggering emotional brain signatures.* Gut Microbes, 2018. **9**(6): p. 486-496.

110. Kivipelto, M., et al., *The Finnish Geriatric Intervention Study to Prevent Cognitive Impairment and Disability (FINGER): study design and progress.* Alzheimers Dement, 2013. **9**(6): p. 657-65.

19. Andrieu, S., et al., *Effect of long-term omega 3 polyunsaturated fatty acid supplementation with or without multidomain intervention on cognitive function in elderly adults with memory complaints (MAPT): a randomised, placebo-controlled trial.* Lancet Neurol, 2017. **16**(5): p. 377-389.

16. Sakurai, T., et al., *Japan-Multimodal Intervention Trial for the Prevention of Dementia: A randomized controlled trial.* Alzheimer's & Dementia, 2024. **20**(6): p. 3918-3930.

17. Zülke, A.E., et al., *A multidomain intervention against cognitive decline in an at-risk-population in Germany: Results from the cluster-randomized AgeWell.de trial.* Alzheimer's & Dementia, 2024. **20**(1): p. 615-628.

111. Sindi, S., et al., *The CAIDE Dementia Risk Score App: The development of an evidence-based mobile application to predict the risk of dementia.* Alzheimers Dement (Amst), 2015. **1**(3): p. 328-33.

160. Brauns, H., S. Scherer, and S. Steinmann, *The CASMIN Educational Classification in International Comparative Research*, in *Advances in Cross-National Comparison: A European Working Book for Demographic and Socio-Economic Variables*, J.H.P. Hoffmeyer-Zlotnik and C. Wolf, Editors. 2003, Springer US: Boston, MA. p. 221-244.

161. UNESCO, I.f.S., *International Standard Classification of Education: ISCED 2011.* 2011: p. 88.

162. Li, R., et al., *Multimodal intervention in older adults improves resting-state functional connectivity between the medial prefrontal cortex and medial temporal lobe†.* 2014. **6**(39).

163. Valls-Pedret, C., et al., *Polyphenol-Rich Foods in the Mediterranean Diet are Associated with Better Cognitive Function in Elderly Subjects at High Cardiovascular Risk.* Journal of Alzheimer's Disease, 2012. **29**: p. 773-782.

164. Hardman, R.J., et al., *Findings of a Pilot Study Investigating the Effects of Mediterranean Diet and Aerobic Exercise on Cognition in Cognitively Healthy Older People Living Independently within Aged-Care Facilities: The Lifestyle Intervention in Independent Living Aged Care (LIILAC) Study.* Current Developments in Nutrition, 2020. **4**(5).

165. Oswald, W.D., et al., *Differential effects of single versus combined cognitive and physical training with older adults: the SimA study in a 5-year perspective.* European journal of ageing, 2006. **3**(4): p. 179-179.

166. Joubert, C. and H. Chainay, *Aging brain: the effect of combined cognitive and physical training on cognition as compared to cognitive and physical training alone - a systematic review.* Clin Interv Aging, 2018. **13**: p. 1267-1301.

167. Sanada, K., et al., *Effects of mindfulness-based interventions on biomarkers in healthy and cancer populations: a systematic review.* BMC Complement Altern Med, 2017. **17**(1): p. 125.

168. Li, Y., et al., *Gut Microbiota Changes and Their Relationship with Inflammation in Patients with Acute and Chronic Insomnia.* Nat Sci Sleep, 2020. **12**: p. 895-905.

169. Bloemendaal, M., et al., *Probiotics-induced changes in gut microbial composition and its effects on cognitive performance after stress: exploratory analyses.* 2021. **11**.

170. Richard, E., et al., *Prevention of dementia by intensive vascular care (PreDIVA): a cluster-randomized trial in progress.* Alzheimer Dis Assoc Disord, 2009. **23**(3): p. 198-204.
